# A Novel Autophagy Inhibitor *p*-Hydroxylcinnamaldehyde Suppresses Esophageal Squamous Cell Carcinoma by Targeting LDHA Phosphorylation-Mediated Metabolic Reprogramming

**DOI:** 10.34133/research.1070

**Published:** 2026-01-12

**Authors:** Sisi Wei, Jingjing Wang, Zhe Zhang, Yuhui Fu, Leyang Zhao, Yanna Bi, Xiaoya Li, Suli Dai, Cong Zhang, Wenjiao Zhu, Li Min, Baoen Shan, Lianmei Zhao

**Affiliations:** ^1^ Research Center, the Fourth Hospital of Hebei Medical University, Shijiazhuang, Hebei 050011, China.; ^2^ Key Laboratory of Tumor Prevention and Precision Diagnosis and Treatment of Hebei, Clinical Oncology Research Center, Shijiazhuang, Hebei 050011, China.; ^3^ Department of Gastroenterology, Beijing Friendship Hospital, Capital Medical University, State Key Laboratory of Digestive Health, National Clinical Research Center for Digestive Diseases, Beijing 100050, China.

## Abstract

Autophagy is integral to the rapid proliferation of esophageal squamous cell carcinoma (ESCC), and its regulation presents a promising avenue for therapeutic intervention. Recent studies have elucidated the interplay between autophagy and glucose metabolism, while there is a paucity of anticancer drugs that concurrently target these 2 biological processes. In this study, we identified a natural compound, *p*-hydroxylcinnamaldehyde (CMSP), originally isolated from Cochinchina momordica seed (CMS) by our research team, which exhibits substantial anticancer activity against ESCC in both in vitro and in vivo models. The study demonstrates that CMSP induces apoptosis in ESCC cell lines and patient-derived organoid (PDO) models by disrupting autophagic flux. Mechanistically, CMSP specifically binds to the glycolytic enzyme LDHA in the cytoplasm, hindering its phosphorylation by blocking its membrane translocation and thereby disrupting its interaction with FGFR1. This inhibition results in decreased lactate production from glycolysis, reduced lysosomal acidity, and suppression of the AMPK/mTOR pathway, ultimately resulting in the blockade of autophagy and the induction of apoptosis. Furthermore, in vivo studies underscore the potential clinical application of CMSP in ESCC by disrupting autophagy. In summary, we propose a novel therapeutic strategy for the precision treatment of ESCC by simultaneously targeting glycolysis-mediated autophagy.

## Introduction

Autophagy is a conserved process where cellular materials are sent to lysosomes for degradation, facilitating nutrient recycling and metabolic adaptation [1–3]. Significantly, elevated autophagy in tumors is essential for supporting tumor proliferation and driving tumor resistance to multiple treatments [4]. Moreover, autophagy and apoptosis are intricately interconnected, with autophagy generally suppressing apoptosis through the inactivation of caspases [5]. Thus, the inhibition of autophagy is reported to potentially restrict tumor growth and enhance the effectiveness of cancer therapies. In esophageal squamous cell carcinoma (ESCC), patient outcomes have been suboptimal due to cancer’s high plasticity and the lack of more efficacious therapeutic strategies [6]. Studies have documented elevated levels of autophagy in ESCC [7–9], implying that targeting this process may offer a promising therapeutic approach.

In anticancer clinical trials, the small-molecule drugs chloroquine (CQ) and hydroxychloroquine (HCQ) are currently the only agents utilized to inhibit autophagy. However, their limited efficacy and the ambiguous mechanisms by which they affect autophagy pose significant challenges to their continued clinical application [2]. Recent efforts have focused on identifying novel autophagy targets, such as small molecular inhibitors of ATG7, ULK1, or ATG4B, which have been designed to inhibit autophagy during the nucleation, elongation, fusion, or degradation stages [10–12]. Nonetheless, despite these advancements, the multifunctional roles of these targets raise questions about the feasibility of identifying highly selective autophagy regulators [13,14]. Evaluating the safety and potential side effects of autophagy inhibitors targeting these pathways remains a critical consideration. Thus, it has been intensified to identify the novel autophagy inhibitors from druggable molecular mechanism.

Recent studies have indicated that some metabolic signals serve as activators to trigger autophagy, and there is emerging evidence suggesting the potential regulation of Warburg effect in the autophagy progression [15–17]. In cancer cells, autophagy could be stimulated by glycolysis through 3 primary mechanisms. Firstly, glycolytic enzymes, such as LDHA [18] and PKM2 [19], are implicated in the initiation of autophagy; these enzymes are frequently overexpressed, undergo posttranslational modifications, or are relocated to cellular compartments beyond the cytosol [20,21]. Secondly, lactate acts as a mediator linking glycolysis to autophagy [22]. Thirdly, 5′-AMP-activated protein kinase (AMPK) serves as a pivotal sensor of cellular stress and energy status, being activated in response to changes in the intracellular adenosine monophosphate (AMP) [or adenosine diphosphate (ADP)] to adenosine triphosphate (ATP) ratio [23,24]. This activation subsequently leads to the suppression of mammalian target of rapamycin (mTOR) and the induction of autophagy, marked by the formation of autophagosomes [25]. Consequently, the AMPK/mTOR signaling pathway plays a vital role in regulation of autophagy, and targeting AMPK offers a promising strategy for developing innovative therapeutic approaches. Collectively, modulating autophagy through metabolic mechanisms may constitute a significant avenue for identification of autophagy inhibitor drugs.

Natural compounds derived from traditional medicine exhibit consideration promise in cancer management due to their multi-target, minimal side effects and favorable safety profile [26]. The present study demonstrates that *p*-hydroxylcinnamaldehyde (CMSP) serves as a novel autophagy inhibitor through regulation of glycolysis by binding to LDHA in ESCC cells. Specifically, CMSP interferes with the pH-dependent fusion of autophagosome and lysosome during the late stage of autophagy, and inactivates the AMPK/mTOR signaling pathways to obstruct the autophagy initiation in the early stage. This dual interference culminates in a lethal autophagic arrest in ESCC cells in vivo and in vitro. Taken together, our findings identify CMSP as a novel and effective autophagy inhibitor with pharmaceutical potential, achieved through the orchestration of a metabolic-to-autophagic signaling cascade.

## Results

### CMSP promotes apoptosis in ESCC cells and patient-derived organoids

**Fig. 1.**
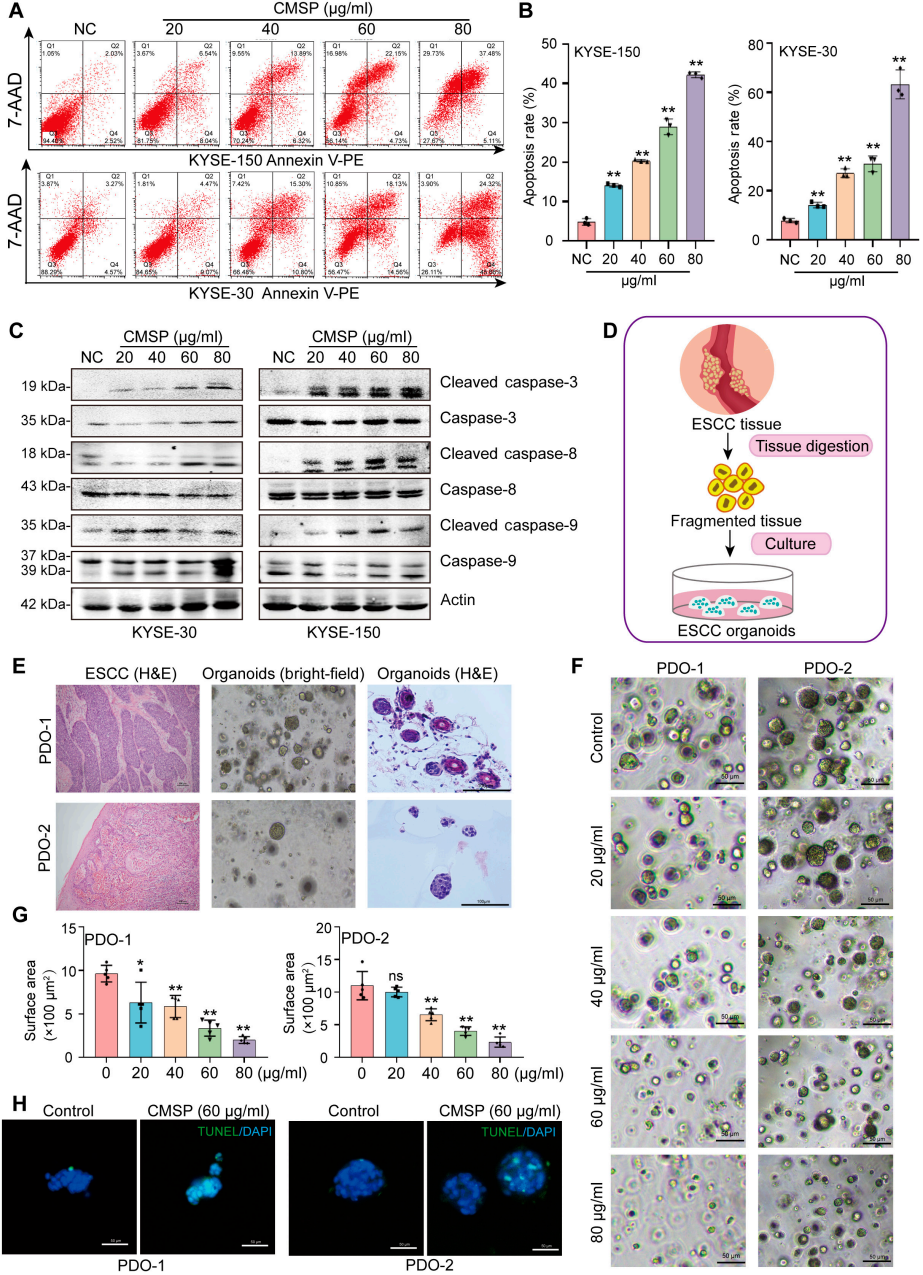
CMSP promotes apoptosis in ESCC cells and organoids. (A) Flow cytometry analysis of KYSE-30 and KYSE-150 cells treated with various concentrations of CMSP (0 to 80 μg/ml) for 36 h. The cells were examined by Annexin V-phycoerythrin (PE)/7-aminoactinomycin D (7-AAD) double staining. The lower right and the upper right quadrants indicate the percentage of apoptotic cells. (B) Statistical analysis of flow cytometry in (A). (C) Western blot analysis of changes in the expression of apoptosis-related proteins in cells treated with CMSP. (D) Overview of organoid construction of ESCC. (E) H&E staining and bright-field images on primary ESCC and/or organoids from ESCC patients. (F) Representative bright-field images of the 2 strains of ESCC organoids treated with different concentrations of CMSP. (G) The surface area was determined 48 h after the coculture with CMSP. (H) The organoids were stained with TUNEL after treatment with CMSP (60 μg/ml for 48 h) or DMSO. The green fluorescence areas indicate apoptosis-positive cells, while the blue 4′,6-diamidino-2-phenylindole (DAPI) staining represents nuclei. Data are shown as the mean ± SD (*n* = 3); one-way ANOVA; *P < 0.05 and **P < 0.01 versus the control group.

CMSP was initially isolated from Cochinchina momordica seed (CMS) by our research team and has been documented to inhibit the viability of ESCC cells at 10 to 40 μg/ml [27,28]. However, the precise mechanism underlying the effect is yet to be clarified. In this study, various concentrations of CMSP were administered to ESCC cell lines and the normal esophageal epithelial cell line HEEC. The results confirmed that CMSP effectively diminished ESCC cell viability in a dose-dependent manner while having modest effect on HEEC proliferation (Fig. S1A and B). Furthermore, flow cytometry analysis consistently demonstrated that CMSP induced apoptosis in ESCC cells in a concentration-dependent manner (Fig. 1A and B). Additionally, increased levels of cleaved caspase-3, caspase-8, and caspase-9 and apoptotic bodies were observed in ESCC cells following CMSP treatment (Fig. 1C and Fig. S1C). These findings indicate that CMSP significantly promotes apoptosis of ESCC cells.

Patient-derived organoids (PDOs) provide valuable platforms for validating potential anticancer drugs for cancers because of the superior ability to capture the characteristics of diseases compared to other in vitro models [29,30]. Thereby, we established PDO models with 3-dimensional structures from freshly dissected ESCC tissues, and both organoid lines exhibited a round morphology lacking any lumen structure, a hallmark of ESCC organoids (Fig. 1D). Further results revealed the histological characteristic of the tumor organoids, closely resembling those observed in ESCC tissues (Fig. 1E). Subsequent analysis indicated that CMSP significantly inhibited the growth and organic conformation of the ESCC organoid strains (PDO-1 and PDO-2) in a dose-dependent manner (Fig. 1F and G). Importantly, the inhibition rate of PDO viability varied significantly according to the specific organoid lineage (Fig. S1D). Consistently, TUNEL (terminal deoxynucleotidyl transferase-mediated deoxyuridine triphosphate nick end labeling) staining results revealed a significantly higher proportion of fluorescein isothiocyanate (FITC)-positive cells, indicative of apoptosis, in the CMSP-treated organoids compared to the control group (Fig. 1H), confirming that CMSP administration induces apoptosis in ESCC organoids.

### CMSP harnesses autophagy and glycolysis in ESCC cells

**Fig. 2.**
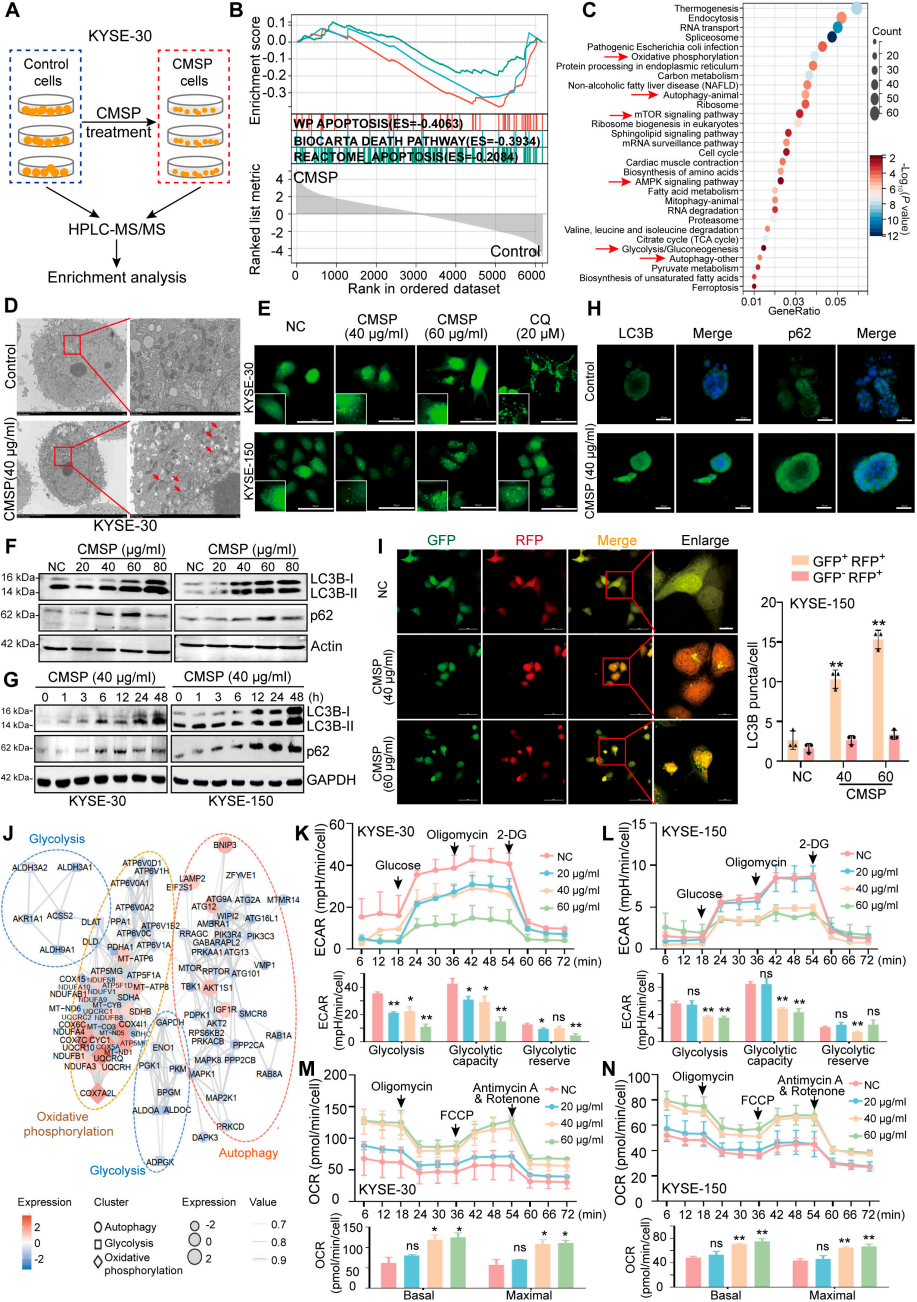
CMSP inhibits autophagy and glycolysis in ESCC cells. (A) Overview of the strategy used in this study. (B) GSEA demonstrating a strong association with apoptosis, following CMSP treatment. (C) Kyoto Encyclopedia of Genes and Genomes (KEGG) analysis for the significantly regulated genes by CMSP compared to control. The top 30 regulated pathways were listed. (D) TEM of KYSE-30 cells treated with CMSP (40 μg/ml) for 36 h. Higher-power magnification of the image of CMSP-treated cells revealed autophagosomes (arrows). (E) Effect of CMSP on GFP-LC3B punctation. (F) Concentration-dependent effect of CMSP on LC3B-II and p62 in KYSE-30 and KYSE-150 after 36 h of treatment. (G) Time-dependent effect of CMSP (40 μg/ml) on LC3B-II and p62. (H) Immunofluorescence of LC3B and p62 on PDO-2 treated with CMSP (60 μg/ml for 48 h) or DMSO. (I) Confocal microscopy showing the yellow LC3B (autophagosome) and red LC3B (autolysosome) dots per cell in each condition of KYSE-150. (J) Protein–protein interaction (PPI) analysis showing the association between autophagy, oxidative phosphorylation, and glycolysis/gluconeogenesis pathways. (K and L) Glycolysis flux was examined by measuring the ECAR using the Seahorse analyzer. The values of glycolysis, glycolytic capacity, and glycolytic reserve were calculated by the Seahorse XF96 software. (M and N) OCR was measured after treatment with various concentrations of CMSP. Basal and maximal OCR levels were at the bottom. Data are shown as the mean ± SD (*n* = 3); Student’s *t* test, one-way ANOVA, or 2-way repeated-measures ANOVA; *P < 0.05 and **P < 0.01 versus the control group.

To gain deeper insights into the molecular mechanisms of CMSP-induced apoptosis, we performed label-free quantitative proteomics on KYSE-30 cells treated with or without CMSP (Fig. 2A). Principal components analysis (PCA) revealed a significant shift in PC1 when comparing the CMSP group to the control group (Fig. S2A). Initial screening identified 1,554 down-regulated proteins and 673 up-regulated proteins in the CMSP group, based on the criteria of |fold change| > 2 and *P* < 0.05 (Tables S1 and S2 and Fig. S2B and C). Congruously, Gene Set Enrichment Analysis (GSEA) indicated a notable enrichment of the apoptosis pathway in the CMSP treatment group (Fig. 2B). Next, significantly altered biological functions enriched by differentially expressed proteins were observed in autophagy, glycolysis, oxidative phosphorylation, and AMPK pathway following CMSP treatment (Fig. 2C and Fig. S2D).

To further dissect the molecular mechanism, we investigated how CMSP influences autophagy level and the progression of glycolysis. Initially, transmission electron microscopy (TEM) revealed a significant rise in the number of autophagic vacuoles relative to control cells (Fig. 2D and Fig. S2E). Furthermore, confocal microscopy analysis corroborated the autophagy-modulating effects of CMSP on ESCC cells transiently expressing green fluorescent protein (GFP)-tagged LC3B, and CMSP treatment preferentially induced an increase in GFP-LC3B puncta (Fig. 2E and Fig. S2F), suggesting that CMSP increased autophagosome of ESCC cells. To further explore the characteristics of intact autophagic flux, preliminary detection of the autophagy marker proteins LC3B and p62 was conducted. Accordingly, our findings revealed a dose- and time-dependent accumulation of LC3B-II in CMSP-treated ESCC cells, accompanied by an elevated protein expression of p62 (Fig. 2F and G). Consistently, the increase in LC3B and p62 levels was observed in ESCC organoids treated with CMSP (Fig. 2H). Collectively, these observations strongly suggest that CMSP treatment suppresses lysosomal degradation in ESCC cells.

Lysosomal acidification and autophagosome–lysosome fusion are essential for the maintenance of functional autophagic flux [31]. Thus, we utilized the tandem fluorescent-tagged LC3B [monomeric red fluorescent protein (mRFP)-GFP-LC3B] plasmid to explore the pH differential between the acidic autolysosome and the neutral autophagosome. Consistent with the aforementioned results, CMSP-treated cells showed a significant increase in the yellow puncta formation without a corresponding rise in the number of red puncta (Fig. 2I and Fig. S2G), reinforcing the conclusion that CMSP inhibits autophagy in ESCC cells by suppressing autophagic flux.

Moreover, the proteomic analysis indicated that CMSP-mediated autophagy is closely associated with the oxidative phosphorylation and glycolysis/gluconeogenesis pathways (Fig. 2J). We next investigated the glycolytic flux by assessing the extracellular acidification rate (ECAR) and oxygen consumption rate (OCR) using the Seahorse Analyzer. The findings revealed that CMSP treatment decreased the overall glycolytic flux (Fig. 2K and L) while simultaneously increasing the oxygen consumption levels (Fig. 2M and N). These results collectively suggest that CMSP simultaneously disrupts autophagic flux and glycolysis in ESCC cells.

### CMSP induces ESCC apoptosis by inhibiting autophagy and glycolysis

**Fig. 3.**
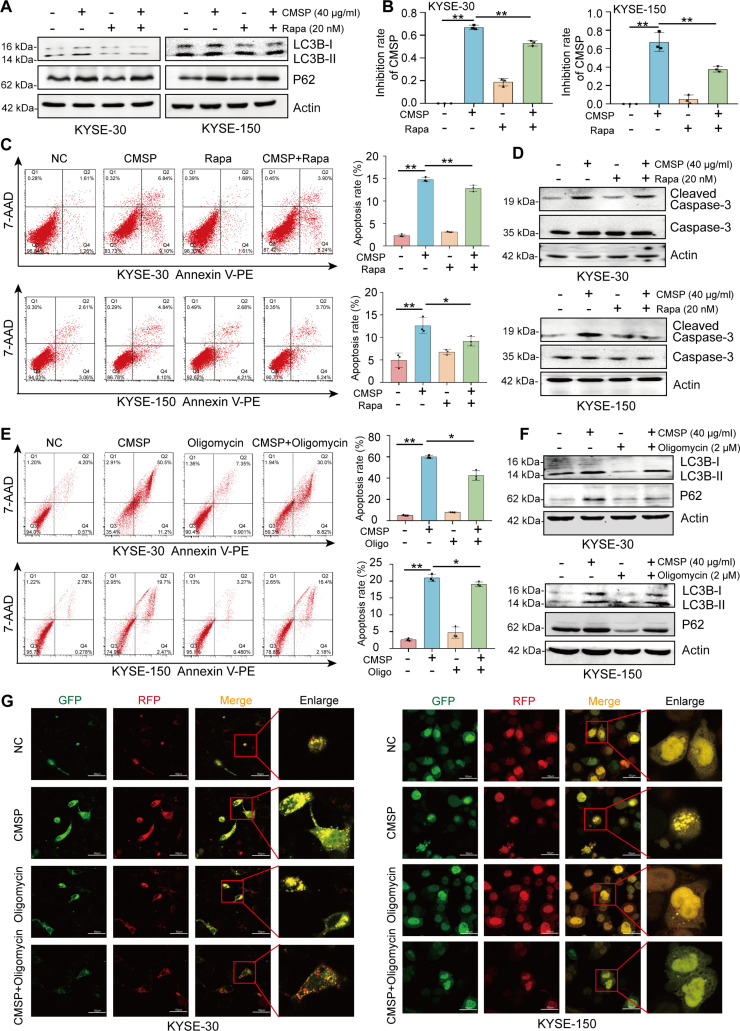
CMSP induces ESCC apoptosis by inhibiting autophagy. (A) Rescue effect of CMSP (40 μg/ml) and autophagy inducer rapamycin (Rapa; 20 nM) on LC3B-II and p62. (B) The MTS [3-(4, 5-dimethylthiazol-2-yl)-5-(3-carboxymethoxyphenyl)-2-(4-sulfophenyl)-2H tetrazolium] assay was performed after treating KYSE-30 and KYSE-150 cells with CMSP (40 μg/ml) or rapamycin (20 nM). (C) Flow cytometry analysis of KYSE-30 and KYSE-150 cells treated with CMSP (40 μg/ml) or rapamycin (20 nM). (D) Western blotting of cleaved caspase-3 treated with CMSP (40 μg/ml) or rapamycin (20 nM). (E) Flow cytometry analysis of KYSE-30 and KYSE-150 cells treated with CMSP (40 μg/ml) or oligomycin (2 μM). (F) Effect of CMSP (40 μg/ml) and oligomycin (2 μM) on LC3B-II and p62. (G) Confocal microscopy showing the yellow LC3B (autophagosome) and red LC3B (autolysosome) dots per cell in each condition of KYSE-30 and KYSE-150. Data are shown as the mean ± SD (*n* = 3); Student’s *t* test; *P < 0.05 and **P < 0.01 versus the control group.

Given the observation that CMSP treatment not only promoted apoptosis but also inhibited autophagy and glycolysis in ESCC cells, we proceeded to dissect the impact of autophagy and glycolysis inhibition in CMSP-induced apoptosis. It is well documented that autophagy and apoptosis interact with each other, with some researchers positing that they are mutually inhibitory, and various cellular molecules enhance apoptosis by suppressing autophagy, leading to a reduction in tumor growth [32,33]. Herein, to further elucidate the involvement of autophagy in CMSP-induced apoptosis, the autophagy activator rapamycin was subsequently employed. Initially, our investigation revealed that the administration of rapamycin notably recovered the levels of LC3B-II and p62, which were predominantly altered by CMSP treatment (Fig. 3A). Subsequently, KYSE-30 and KYSE-150 cells subjected to CMSP exhibited a notable increase in proliferation inhibition, an effect that was considerably diminished when rapamycin was used alongside CMSP (Fig. 3B). Congruously, flow cytometry and cleaved caspase-3 analysis demonstrated that CMSP enhanced apoptosis in ESCC cells, which was significantly reversed by the co-administration of rapamycin (Fig. 3C and D).

Additionally, oligomycin was employed as a glycolysis stimulator to elucidate the role of glycolysis in CMSP-induced apoptosis [34]. Flow cytometry analysis indicated that oligomycin partially rescued the apoptosis induced by CMSP (Fig. 3E). Furthermore, oligomycin treatment remarkably restored the levels of LC3B-II and p62, which were primarily changed by CMSP treatment (Fig. 3F). Similar results were observed where CMSP-induced inhibition of autophagic flux was partially reversed with oligomycin treatment (Fig. 3G). Collectively, the data show that CMSP promotes apoptosis in ESCC by inhibiting both autophagy and glycolysis, while activation of glycolysis can counteract the CMSP-induced inhibition of autophagy.

### CMSP directly binds to LDHA and blocks its phosphorylation through disconnection with FGFR1

**Fig. 4.**
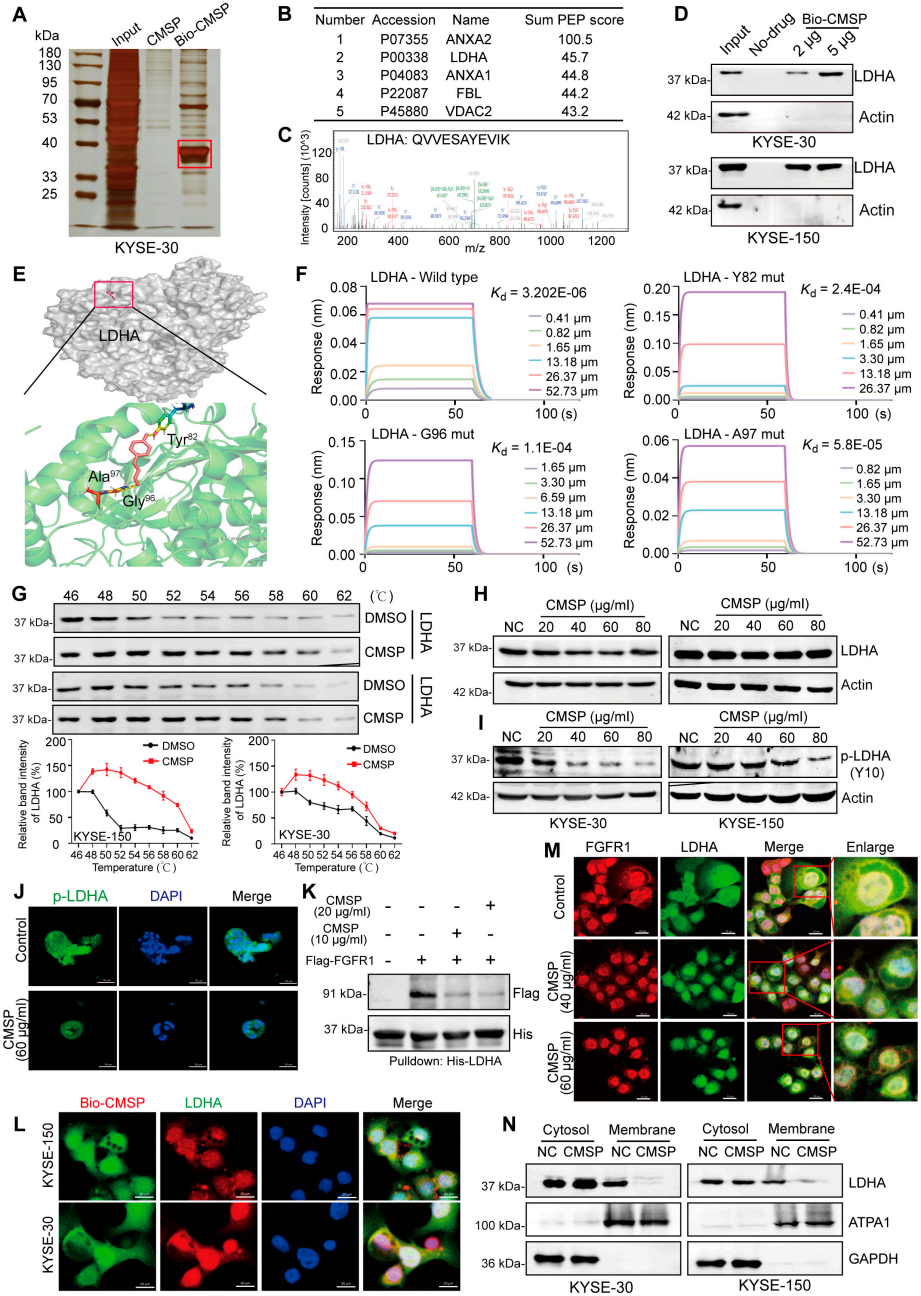
CMSP directly binds to LDHA and inhibits the phosphorylation through disconnection with FGFR1. (A) Representative image of silver-stained SDS-PAGE used for the separation of proteins enriched by biotin-CMSP. Specific bands used for further MS identification were assigned red frame. (B) Top 5 binding proteins of the CMSP. (C) Amino acid sequence of LDHA as detected by MS. (D) Validation of the interaction between LDHA and CMSP. (E) Predicted interaction between CMSP and LDHA. LDHA (PDB ID: 4ZVV) was presented (labeled in light green and rendered as ribbons). The sites of CMSP (stick rendering) binding to target subunit via a predicted hydrogen bond were shown. (F) Binding affinity of CMSP to recombinant LDHA and mutation protein was evaluated by BLI assay. (G) CETSA assay was used to evaluate the binding between CMSP and LDHA in thermodynamic levels. (H) Western blot of LDHA following treatment of various concentrations of CMSP. (I) Representative Western blot of p-LDHA following treatment of concentration-dependent CMSP for 36 h. (J) Immunofluorescence of p-LDHA on PDO-2 treated with CMSP (60 μg/ml for 48 h) or DMSO. (K) His-tag pull-down assay results showing that CMSP inhibits the binding between FGFR1 and LDHA. (L) Immunofluorescence of Bio-CMSP and LDHA on KYSE-30 and KYSE-150 treated with Bio-CMSP (80 μg/ml for 36 h) or DMSO. (M) Immunofluorescence of FGFR1 and LDHA on KYSE-150 treated with CMSP (40 or 60 μg/ml for 36 h) or DMSO. (N) Immunoblots of LDHA in the cytosol and membrane fractions. GAPDH was examined as the cytosol marker, and ATPA1 was used as the membrane marker.

To elucidate the underlying mechanisms by which CMSP induces apoptosis in ESCC cells, we synthesized a biotin-labeled probe (biotin-CMSP) to investigate the specific proteins interacting with CMSP (Fig. S3A and B). The pull-down assay revealed a distinct band in the biotin-CMSP group (Fig. 4A), and subsequent high-performance liquid chromatography–tandem mass spectrometry (HPLC-MS/MS) analysis identified 516 proteins potentially binding to CMSP (Table S3). Notably, the glycolytic enzyme LDHA was among the top 5 enriched binding proteins (Fig. 4B and C). Further pull-down/Western blot assay confirmed LDHA protein as a potential target (Fig. 4D). As illustrated in Fig. S3C, LDHA expression was significantly elevated in ESCC samples compared to normal controls, according to public data from UALCAN (https://ualcan.path.uab.edu/) and GEPIA (https://gepia.cancer-pku.cn/). Moreover, LDHA is recognized for its crucial role in the Warburg effect; thus, we hypothesize that it may explain the molecular mechanisms on how CMSP inhibits glycolysis in ESCC cells. Furthermore, molecular docking and dynamics simulation indicated that CMSP interacted with LDHA protein, likely through the residues Tyr^82^, Gly^96^, and Ala^97^ (Fig. 4E). To confirm the computational prediction, we purified LDHA protein, as well as the LDHA^Y82mut^, LDHA^G96mut^, and LDHA^A97mut^ proteins, in which Tyr^82^, Gly^96^, and Ala^97^ were mutated, respectively (Fig. S3D and E). The results from biolayer interferometry assay (BLI) showed that CMSP can bind to LDHA with a *K*_d_ of 3.202 × 10^−6^, which was disrupted when Tyr^82^, Gly^96^, or Ala^97^ of LDHA was mutated (*K*_d_^Y82mut^ = 2.4 × 10^−4^, *K*_d_^G96mut^ = 1.1 × 10^−4^, *K*_d_^A97mut^ = 5.8 × 10^−5^) (Fig. 4F and Fig. S3F). These findings provided direct evidence that CMSP preferentially binds to the LDHA wild-type rather than the mutants, suggesting the crucial role of Tyr^82^, Gly^96^, or Ala^97^ in this binding process. Subsequently, we employed the cellular thermal shift assay (CETSA) to reveal that CMSP enhanced the thermotolerance of LDHA within a temperature range of 46 to 62 °C (Fig. 4G). These findings suggested that CMSP specifically binds to LDHA.

However, our further results indicated that CMSP did not alter the overall expression level of LDHA (Fig. 4H). Given that studies have suggested that direct phosphorylation at Y10 could increase LDHA’s enzymatic activity [35], we found that CMSP treatment notably decreased this phosphorylation (Fig. 4I), consistent with this hypothesis. Similarly, a decrease in phosphorylated LDHA was also observed in ESCC organoids treated with CMSP (Fig. 4J). Additionally, it has been documented that the phosphorylation of LDHA is regulated by FGFR1, a membrane receptor tyrosine kinase frequently overexpressed in various cancers [36]. Then, we conducted an analysis of FGFR1 and p-LDHA expression in ESCC tissue microarrays. The findings indicated a significant overexpression of FGFR1 and p-LDHA in ESCC tissues compared to normal tissues, with a notable positive relationship between FGFR1 and p-LDHA expression in the ESCC samples (Fig. S4A). Subsequently, a His-tag pull-down assay confirmed the direct interaction between recombinant LDHA (rLDHA) and FGFR1 (rFGFR1), and the presence of CMSP attenuated this interaction (Fig. 4K), although CMSP did not influence FGFR1 expression levels, nor was there any detectable interaction between CMSP and FGFR1 (Fig. S4B and C).

Considering that FGFR1 is a membrane-associated protein, we postulated that CMSP might influence the intracellular distribution of LDHA, thereby inhibiting FGFR1-induced phosphorylation at the membrane. Initially, both LDHA and CMSP were observed to be localized in the cytoplasm (Fig. 4L). Subsequent confocal microscopy revealed that FGFR1 and LDHA were colocalized on the cell membrane. However, upon the addition of CMSP, this colocalization was reduced (Fig. 4M). Consistently, subcellular fractionation analysis confirmed that CMSP treatment led to a decrease in LDHA’s membrane localization (Fig. 4N). Taken together, these findings imply that CMSP interacts with LDHA intracellularly, disrupting its membrane localization and competitively inhibiting FGFR1’s binding to the enzyme, thereby impeding its phosphorylation activity.

### CMSP inhibits autophagy by impeding glycolysis-mediated lysosomal acidification

**Fig. 5.**
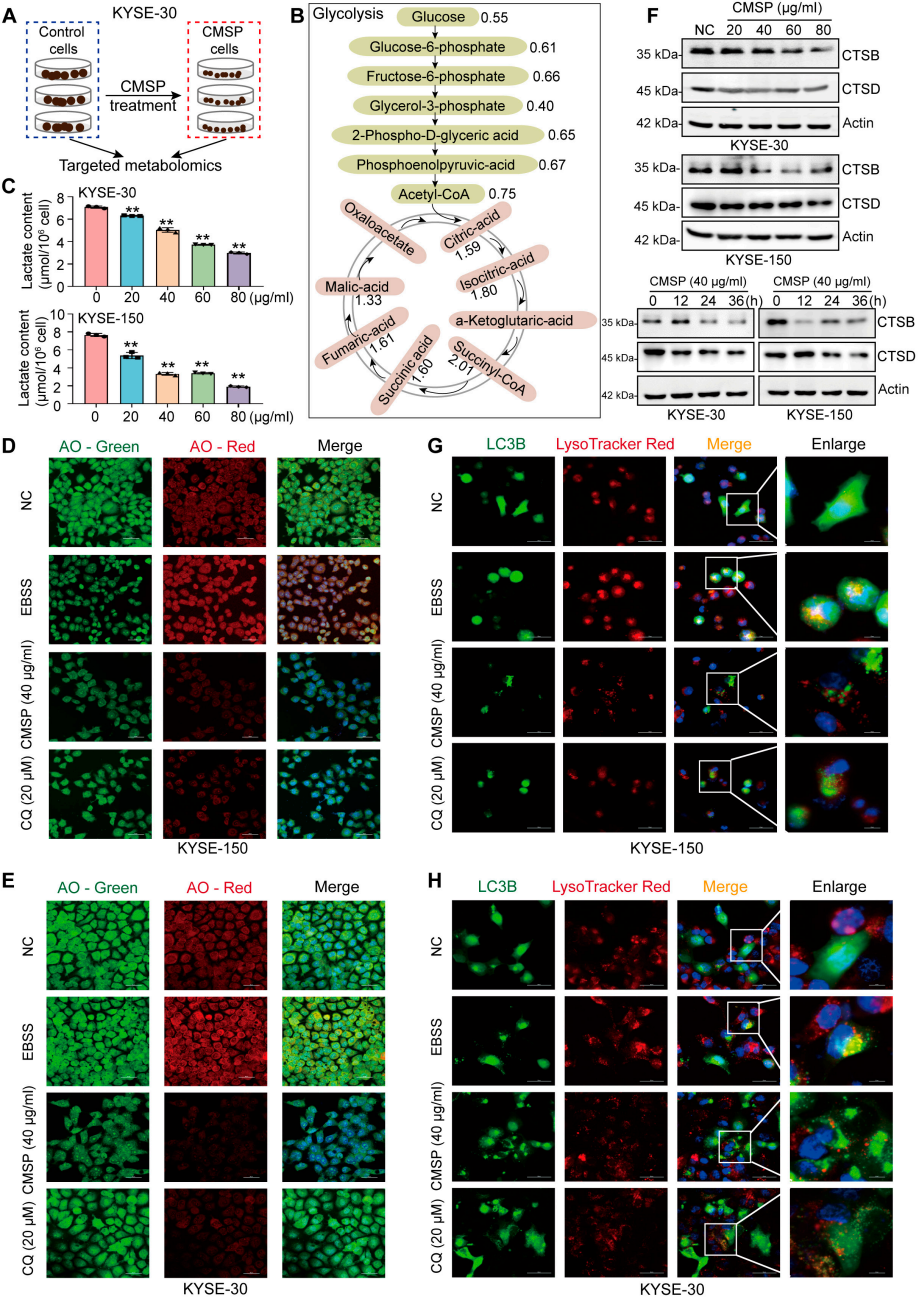
CMSP inhibits autophagy by impeding lysosomal acidification. (A) Overview of the targeted metabolomics used in this study. Control and CMSP-treated group, *n* = 3 independent experiments. (B) Overview of the glycolysis. (C) Lactate content in cell culture supernatant of KYSE-30 and KYSE-150 cells under various concentrations of CMSP (0 to 80 μg/ml) for 36 h. (D and E) CMSP affects AO staining of acidic compartments. KYSE-150 (D) and KYSE-30 (E) cells were incubated with or without CMSP and CQ or starved in EBSS for 12 h before adding AO. (F) Western blotting analysis for the expression of endogenous CTSB and CTSD across various concentrations and time points. (G and H) Effect of CMSP on autophagosome maturation. Confocal microscopy showing the colocalization of LC3B and lysosome in KYSE-150 and KYSE-30. Data are shown as the mean ± SD (*n* = 3); one-way ANOVA; **P < 0.01 versus the control group.

Considering the pivotal roles of LDHA in aerobic glycolysis, it is plausible to hypothesize that CMSP’s interaction with glycolytic enzymes have an effect on the glycolytic metabolic pathway in the cells. Thus, targeted metabolomics was employed to assess changes in central carbon metabolites following CMSP treatment (Fig. 5A). In accordance with the findings, the concentrations of most intermediates involved in glycolytic pathways were generally reduced, whereas those associated with the tricarboxylic acid (TCA) cycle were elevated under CMSP treatment (Fig. 5B and Fig. S5A). Subsequent analysis revealed the lactate content within ESCC cells. The results demonstrated a decrease in lactate production in both KYSE-30 and KYSE-150 cells following treatment with CMSP (Fig. 5C), thereby confirming that CMSP effectively inhibits glycolysis and the subsequent production of lactate in ESCC cells.

A rising number of findings point to lactate’s role in facilitating lysosomal acidification and autophagy in cancer cells [21,37], implying that CMSP might influence autophagic activity by modulating lactate level derived from glycolytic metabolism. Acridine orange (AO) exhibits green fluorescence in cytoplasmic structures and red fluorescence in the intact acidic lysosome environment. Therefore, it can be an indicator for detecting changes in lysosomal acidity and integrity. Accordingly, the red fluorescence was increased in the Earle’s balance salt solution (EBSS)-treated ESCC cells (positive control) and markedly decreased in cells exposed to CMSP and CQ (Fig. 5D and E), suggesting that lysosomal pH is increased upon CMSP treatment. Subsequently, we evaluated the impact of CMSP treatment on the expression levels of lysosomal enzymes cathepsin B (CTSB) and cathepsin D (CTSD) to ascertain that CMSP affects lysosomal functionality. The results revealed that CMSP significantly diminished the protein expression levels of CTSB and CTSD in a dose-dependent manner and on indicated time points (Fig. 5F). To further verify the characteristics of lysosomal function, ESCC cells expressing GFP-LC3B were stained with LysoTracker, a lysosome-specific dye to detect the colocalization of autophagosome and lysosome. As a positive control, starvation with EBSS significantly enhanced the colocalization of GFP-LC3B puncta with LysoTracker-stained subregions. In contrast, this colocalization was disrupted in the presence of CMSP or CQ (Fig. 5G and H). Collectively, these findings indicate that CMSP treatment substantially inhibited autophagy in ESCC cells by impeding lysosomal acidification.

### CMSP reduces glycolysis-regulated AMPK/mTOR pathway activity in ESCC cells

**Fig. 6.**
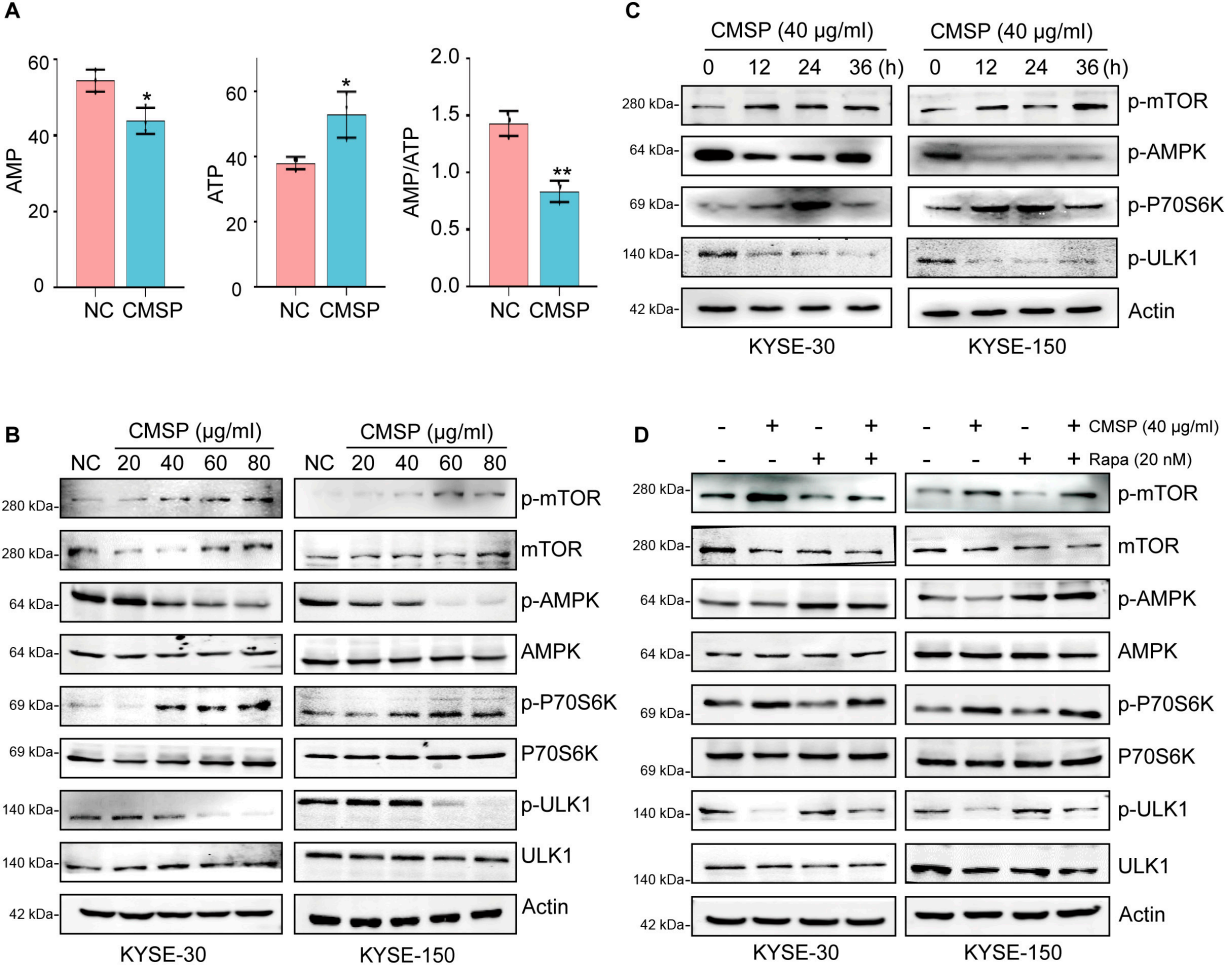
CMSP reduces AMPK/mTOR pathway activity in ESCC cells. (A) AMP, ATP, and ratio of AMP and ATP response to CMSP treatment in targeted metabolomics. (B) Representative Western blot of p-mTOR, mTOR, p-AMPK, AMPK, p-P70S6K, P70S6K, p-ULK1, and ULK1 following treatment of concentration-dependent CMSP for 36 h. (C) Representative Western blot of p-mTOR, p-AMPK, p-P70S6K, and p-ULK1 following treatment of CMSP (40 μg/ml) for different time. (D) Representative Western blot of p-mTOR, p-AMPK, p-P70S6K and p-ULK1 following treatment of CMSP (40 μg/ml) and rapamycin (20 nM). Data are shown as the mean ± SD (*n* = 3); Student’s *t* test; **P < 0.01 versus the control group.

Subsequently, we explored the detailed molecular mechanism underlying CMSP’s inhibition of autophagy through regulation of glycolysis. It is well established that activation of AMPK is known to inhibit mTOR phosphorylation, thereby initiating autophagy [38]. In addition, AMPK is responsible for regulating cellular energy homeostasis [39]. Thus, we reasoned that CMSP inhibited autophagy through glycolysis-mediated AMPK/mTOR pathway regulation. First, our findings indicated that CMSP-mediated inhibition of glycolysis and enhancement of oxidative phosphorylation resulted in a decreased AMP/ATP ratio and reduced lactate production in tumor cells (Fig. 6A). Additionally, treatment of CMSP effectively decreased the activation of p-AMPK (Thr^172^) in a dose- and time-dependent manner in KYSE-30 and KYSE-150 cells. In accordance with this, the downstream target of p-AMPK, p-ULK1, exhibited a similar trend, whereas the expression levels of p-mTOR and p-P70S6K demonstrated a significant increase (Fig. 6B and C). However, no significant impact was observed on the overall expression levels of AMPK, ULK1, mTOR, and P70S6K (Fig. S5B and C). Moreover, treatment with rapamycin reversed the expression changes of these proteins, providing further evidence that CMSP inhibits the AMPK signaling pathway (Fig. 6D and Fig. S5D). Collectively, our findings indicate that CMSP inhibits autophagy in ESCC cells through the glycolysis-regulated AMPK/mTOR pathway.

### CMSP suppresses autophagy, thereby inducing ESCC apoptosis in vivo

**Fig. 7.**
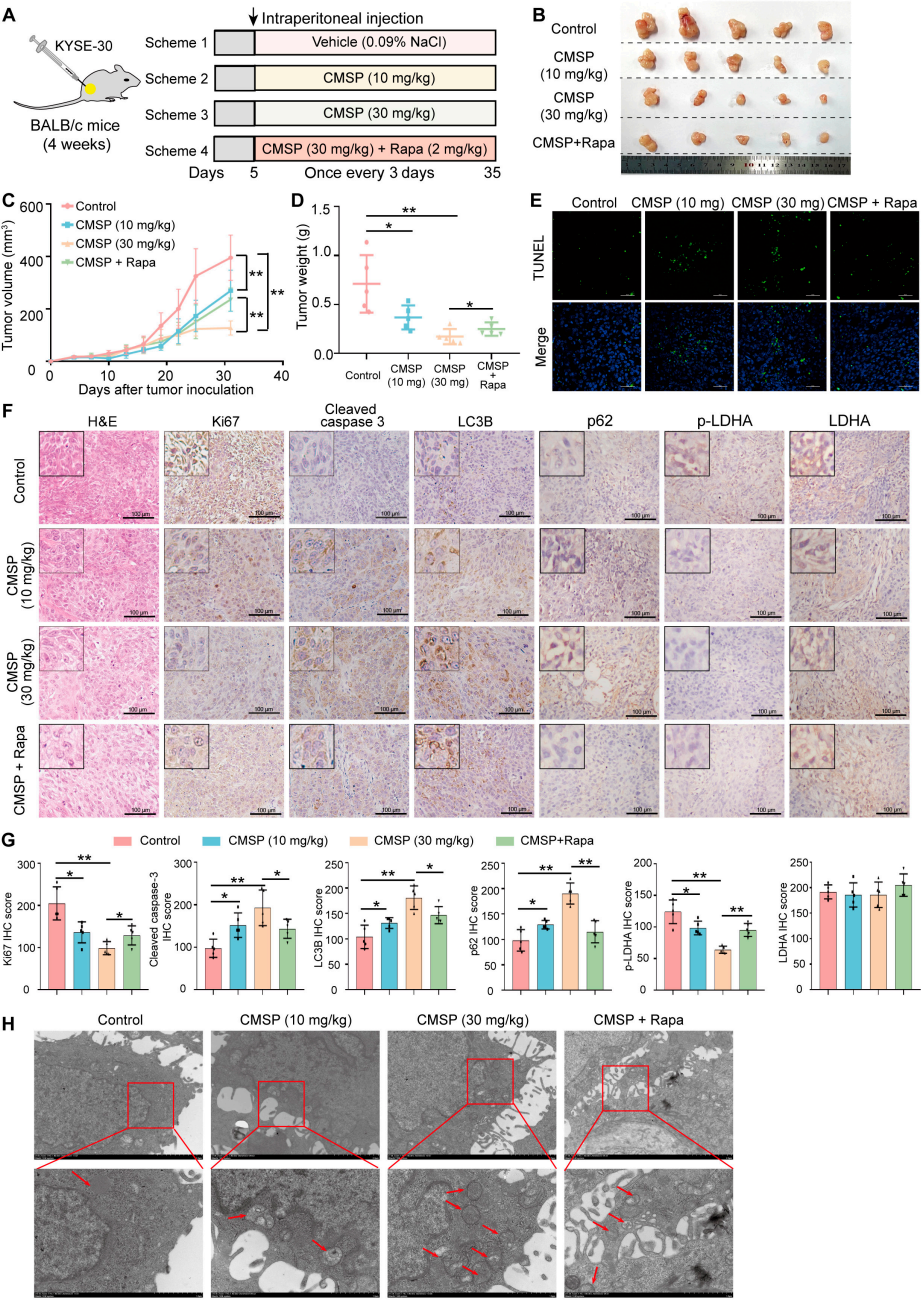
CMSP induces ESCC apoptosis by inhibiting autophagy in vivo*.* (A) Schematic presentation of the experimental protocol. (B) Representative pictures of tumors. *n* = 5 per group. (C and D) Tumor volume and weight of mice from each group. (E) The tumors were stained with TUNEL. The green fluorescence areas indicate apoptosis-positive cells, while the blue DAPI staining represents nuclei. (F) Tumor biopsies were stained with H&E, Ki67, LC3B, p62, cleaved caspase-3, p-LDHA, and LDHA. (G) Statistics of Ki67, LC3B, p62, cleaved caspase-3, p-LDHA, and LDHA IHC in (E). (H) TEM of tumor biopsies in different groups. Data are shown as the mean ± SD (*n* = 5); one-way ANOVA or Student’s *t* test; *P < 0.05 and **P < 0.01 versus the control group.

The above results demonstrated that CMSP induces ESCC apoptosis by inhibiting cell autophagy. Next, to investigate the inhibition function of CMSP in vivo, we developed an ESCC xenograft tumor model using BALB/c mice (Fig. 7A). After 35 d of treatment, xenograft tumors treated with CMSP (10 or 30 mg/kg) exhibited a significant reduction in weight and volume compared to the control group, while this effect was partially reversed by the combination therapy of rapamycin and CMSP (Fig. 7B to D), suggesting that the therapeutic effect of CMSP may occur through the inhibition of autophagy. Subsequent TUNEL staining results demonstrated a significantly increased proportion of FITC-positive cells, indicative of apoptosis, in the CMSP-treated group. This effect was reversed by rapamycin therapy, suggesting that CMSP induces apoptosis in ESCC through the inhibition of autophagy (Fig. 7E). Furthermore, immunohistochemical (IHC) assays were conducted on xenografted tumor sections to assess Ki67, LC3B, p62, cleaved caspase-3, p-LDHA, and LDHA expression. Following CMSP therapy, the treatment group exhibited a significant decrease in Ki67 levels, indicating a suppression of cancer cell proliferation. Additionally, tumor tissues in the CMSP treatment group exhibited elevated expression levels of LC3B, p62, and cleaved caspase-3, alongside a reduction in p-LDHA expression, when compared to the control group. These molecular alterations were reversed upon the combined administration of CMSP and rapamycin. Notably, the expression of LDHA remained unchanged (Fig. 7F and G). These observations were consistent with the results obtained from the immunoblotting assay. Furthermore, TEM analysis of xenografted tumor sections revealed an accumulation of autophagosomes following CMSP treatment (Fig. 7H). To further assess the potential toxicity of CMSP in vivo, subsequent experiments were conducted. The findings indicated no significant variations in body weight across the various mouse groups (Fig. S6A). Serum levels of aspartate aminotransferase (AST), alanine aminotransferase (ALT), UREA, and creatinine (CREA) were comparable across all groups (Fig. S6B). Additionally, hematoxylin and eosin (H&E) analysis of major organs showed no significant liver and kidney damage, lung toxicity, or inflammatory infiltration in the spleen within the CMSP group (Fig. S6C). Collectively, these findings indicate that CMSP effectively inhibits the progression of ESCC and induces apoptosis by suppressing autophagy in vivo while demonstrating a favorable safety profile.

**Fig. 8.**
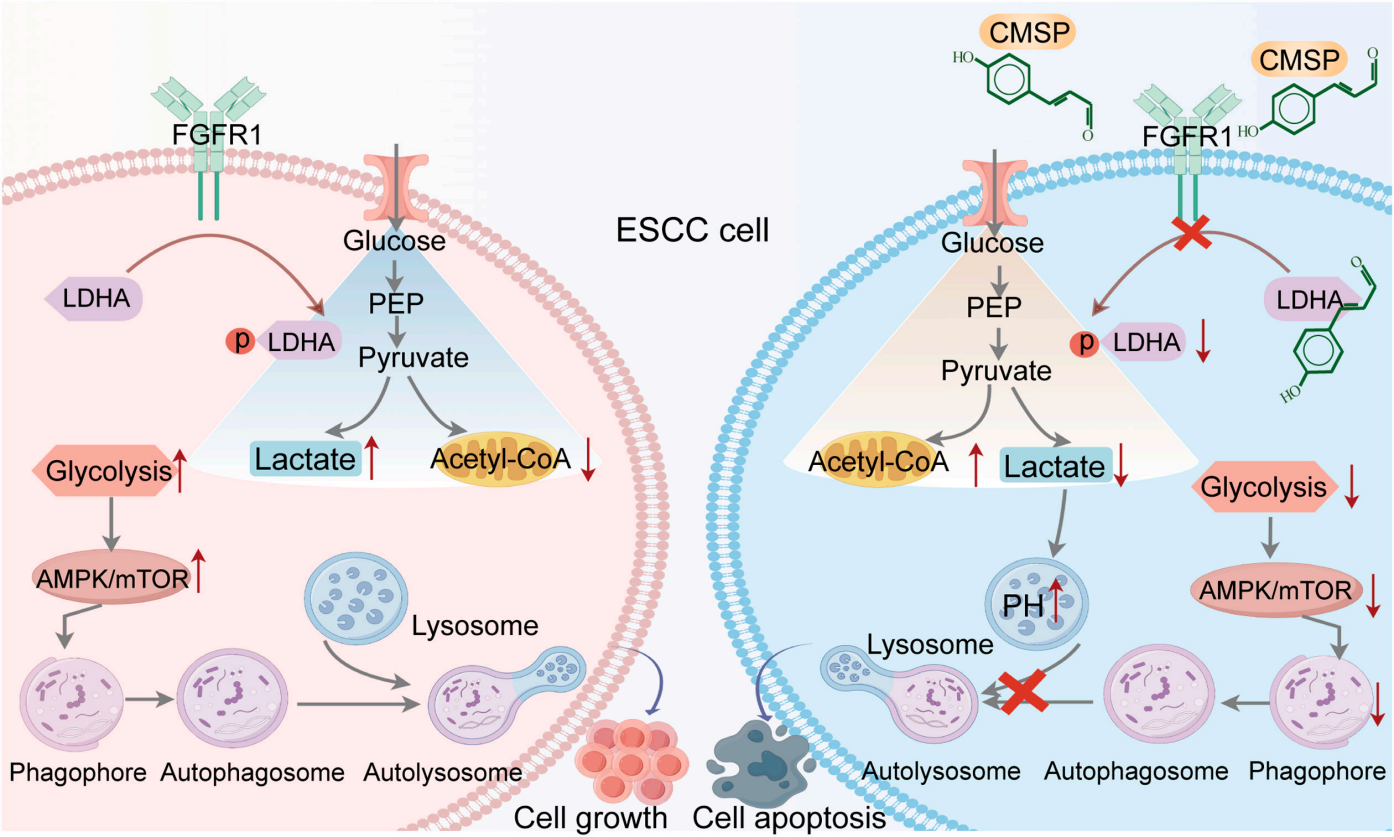
Schematic of molecular mechanisms. The proposed molecular mechanism by which CMSP promotes cell apoptosis by inhibiting glycolysis and cell autophagy.

In conclusion, our cumulative findings demonstrate that CMSP specifically binds to the glycolytic enzyme LDHA intracellularly, inhibiting its phosphorylation by preventing its translocation to membrane for interaction with FGFR1. This leads to a reduction in glycolysis and an enhancement of oxidative phosphorylation in ESCC, resulting in decreased AMP/ATP ratios and lactate production. Consequently, CMSP suppresses autophagy in ESCC cells by reducing lysosomal acidification and the AMPK/mTOR pathway, ultimately triggering apoptosis in tumor cells (Fig. 8).

## Discussion

Throughout the past decade, autophagy has attracted considerable attention for its potential in developing novel therapeutic approaches [40]. Consequently, inhibition of autophagy is considered a promising cancer treatment strategy, either by promoting autophagy-related cell death or by enhancing tumor cell sensitivity to drug-induced apoptosis [41–43]. Nonetheless, the mechanisms regulating autophagy and apoptosis in ESCC remain largely unexplored. Recent studies have identified a few natural compounds that can efficiently inhibit autophagy [44,45], indicating that natural products may be potential sources for the development of new autophagy inhibitors. Herein, the study represents the first to identify that CMSP can concurrently inhibit both glycolysis and autophagy with a dual-targeting mechanism by specifically binding to LDHA, thus eliciting a powerful antitumor effect. Moreover, the study novelly reveals that LDHA not only functions as a pivotal enzyme in glycolysis but also modulates autophagy through its membrane translocation and interaction with FGFR1. This introduces a novel strategy for the precise treatment of ESCC.

Our research team’s prior investigations indicated that CMSP concentrations below 40 μg/ml attenuate the malignancy of ESCC cells by inhibiting cell invasion, migration, and colony formation ability [27,28]. In this study, we identified that CMSP concentrations ranging from 40 to 80 μg/ml markedly enhance apoptosis in a dose-dependent manner, highlighting the critical importance of the concentration range for optimizing CMSP’s therapeutic efficacy. Furthermore, our findings demonstrated that CMSP effectively suppressed autophagy in both concentration- and time-dependent ways, as evidenced by increased expression of LC3B-II and p62. The up-regulation of LC3B-II, a typical autophagosomal biomarker, in the CMSP-treated groups suggests an increase in autophagosome quantity, corroborated by fluorescence microscopy findings. Furthermore, the accumulation of p62, a specific substrate that is recruited to the autophagosomal membrane and subsequently degraded in autolysosomes [46], was observed in CMSP-treated groups, indicating an impediment in lysosomal degradation processes. Consequently, CMSP has the potential to disrupt autophagic flux and exacerbate the accumulation of aberrant autophagosomes. Notably, the effects obtained with CMSP were comparable to those observed with CQ, a recognized autophagosome–lysosome fusion inhibitor.

To elucidate the therapeutic targets of CMSP, it is essential to investigate the proteins that interact with it, a significant finding of this study. A pull-down assay, followed by silver staining and LC-MS analysis, identified 516 candidate targets for CMSP. In Fig. 2J to M, we have confirmed that CMSP treatment results in a reduction of the overall glycolytic flux while concurrently enhancing oxygen consumption levels. Among the top 5 CMSP-binding proteins, only LDHA is a classical glycolytic enzyme. The top-ranked protein, ANXA2, is a prominent member of the calcium-dependent phospholipid-binding protein family and is integral to physiological processes such as cell membrane transport, signal transduction, and cytoskeleton reorganization. However, few studies indicate that ANXA2 directly regulates glucose metabolism. Consequently, LDHA was selected for further analysis. Cancer is characterized by alterations in cellular metabolism, with tumor cells frequently exhibiting elevated glucose consumption and lactate production. This metabolic alteration facilitates biosynthesis, thereby sustaining tumor growth [47,48]. Recent studies suggest that certain antineoplastic compounds derived from natural sources exhibit regulatory effects on glucose metabolism, with enzymes potentially serving as their targets [49]. For instance, DT-13, a saponin monomer extracted from *Liriopes Radix*, has been reported to inhibit glucose uptake, ATP generation, and lactate production by significantly suppressing GLUT1 expression in human colorectal cancer cells [50].

In addition, our observations indicate a decrease in the phosphorylation levels of LDHA following CMSP treatment. LDHA, an enzyme responsible for catalyzing the conversion of pyruvate to lactate, is positively associated with the Warburg effect [51,52]. Various posttranslational modifications, such as phosphorylation and acetylation, regulate its enzymatic activity [52,53]. FGFR1, an oncogenic receptor tyrosine kinase, directly phosphorylates LDHA, thereby enhancing the interaction between LDHA and its substrate NADH [reduced form of nicotinamide adenine dinucleotide (oxidized form)] and increasing LDHA’s enzymatic activity [52]. It has been reported that FGFR1-mediated phosphorylation of LDHA at the Y10 site could drive lactate production [54,55]. However, few studies investigate the natural compounds to inhibit FGFR1-mediated LDHA phosphorylation. Moreover, previous research has demonstrated that patients with ESCC frequently exhibit significant amplification of the FGFR1 gene, a condition that is associated with a poorer prognosis [56]. As a result, CMSP may exert a more pronounced therapeutic effect on patients with FGFR1 amplification.

Another important finding from our study is the decreased lactate production following CMSP treatment. Recent studies have indicated that an acidic tumor microenvironment is prevalent in various cancers, mainly resulting from lactate production via the Warburg effect [57,58]. The excretion of lactate is pivotal in maintaining intracellular pH homeostasis while concurrently acidifying the extracellular environment. This acidic environment modulates cell proliferation and promotes metastasis, invasion, angiogenesis, and poor prognosis [59–61]. In this study, a reduction in lactate levels resulted in an alleviation of lysosomal acidification, which is crucial for the optimal activity of hydrolases. Lactate functions as a signaling molecule connecting glycolysis with autophagy. Firstly, lactate molecules could covalently modify TFEB, leading to its lactylation and resulting in increased TFEB activity and autophagy flux [62]. Additionally, elevated lactate levels have been reported to mediate PIK3C3/VPS34 lactylation and induce autophagy [22]. It is hypothesized that the reduction of lactate impairs lysosomal functions during the autophagy process, ultimately obstructing autophagic flux. We have not yet determined whether core autophagy proteins undergo lactylation following treatment with CMSP. This novel molecular mechanism warrants further investigation, and we plan to explore this issue in future studies.

Autophagy is modulated by numerous molecules. Our study focused on the AMPK/mTOR pathway, a well-established regulatory mechanism. AMPK is crucial in autophagy regulation as it maintains cellular energy homeostasis. Consequently, small molecules that inhibit autophagy through the AMPK pathway hold significant therapeutic promise for impeding the progression of ESCC. Notably, CMSP treatment in KYSE-30 and KYSE-150 cell lines led to a decrease in AMPK phosphorylation at Thr^172^. Additionally, the inhibition of autophagy was confirmed by mTOR activation and decreased ULK1 phosphorylation, both in a dose- and time-dependent way.

In summary, the findings from our study elucidate the intricate interactions among CMSP, glycolysis, autophagy, and apoptosis in tumor cells. We have demonstrated for the first time that CMSP induces apoptosis in ESCC cells by disrupting autophagic flux through its interaction with LDHA. This interaction results in a reduction in glycolysis, suppression of lactate production, and inhibition of the AMPK/mTOR pathway, thereby inhibiting cellular autophagy. Our results suggest that autophagy-modulating small molecules, CMSP, have the potential to serve as effective therapeutic agents in cancer treatment. Furthermore, targeting glycolysis may offer a promising therapeutic strategy.

## Materials and Methods

### Cell lines and cell culture

ESCC cell lines KYSE-30, KYSE-150, KYSE-450, and YES-2, along with the normal esophageal epithelial cell line HEEC, were cultured in RPMI 1640 medium (Gibco, USA), with 10% fetal bovine serum (BI, Israel) and 1% penicillin/streptomycin (Invitrogen, USA). The cultures were incubated at 37 °C in a humidified environment containing 5% CO_2_. These cell lines were obtained from Pricella, and their authenticity was confirmed through short tandem repeat (STR) profiling.

### Reagents and antibodies

Antibodies against cleaved caspase-3 (#9664), caspase-3 (#9662), cleaved caspase-8 (#9496), caspase-8 (#4790), cleaved caspase-9 (#9505), caspase-9 (#9508), p-AMPK (#2535), and p-ULK1 (#14202) were sourced from Cell Signaling Technology (Beverly, MA, USA). Antibodies against actin (20536-1-AP), LC3B (14600-1-AP), glyceraldehyde-3-phosphate dehydrogenase (GAPDH) (10494-1-AP), p-mTOR (67778-1-Ig), mTOR (66888-1-Ig), AMPK (66536-1-Ig), P70S6K (14485-1-AP), and ULK1 (20986-1-AP) were purchased from Proteintech Group (Wuhan, China). Antibody against p-P70S6K (AF3228) was purchased from Affinity Biosciences (Cincinnati, OH, USA). Antibody against ATPA1 (380790) was purchased from ZEN-BIOSCIENCE (Chengdu, China). Antibody against CTSB (PTM-6372) was purchased from PTM BIO (Hangzhou, China). Antibody against CTSD (sc-377299) was purchased from Santa Cruz Biotechnology (Santa Cruz, CA, USA).

CQ (IC4440) and EBSS (H2045) were purchased from Solarbio. Rapamycin (HY-10219) was purchased from MedChemExpress. LysoTracker Red (C1046) was purchased from Beyotime. Plasmids encoding GFP-LC3B and RFP-GFP-LC3B were obtained from Youbio (Changsha, China). Plasmids encoding His-LDHA, LDHA mutants, and flag-FGFR1 were obtained from Sangon Biotech (Shanghai, China). Lipofectamine 2000 was obtained from Invitrogen (Shanghai, China).

### Isolation and extraction of CMSP

CMSs were procured from Lerentang Pharmacy in Shijiazhuang and authenticated by R. Fengzhi. The voucher specimen, identified as No.2016-NCPC-224, is preserved at North China Pharmaceutical Group Corporation in Shijiazhuang. Following our previously established protocol [63], CMSP was successfully isolated, confirmed endotoxin-free, and stored in dimethyl sulfoxide (DMSO) at 100 mg/ml with at least 95% purity. Prior to experimental use, CMSP was diluted in a serum-free medium to achieve the desired concentration, keeping DMSO below 0.01%.

### ESCC PDO culture

ESCC organoids were successfully established at BioGenous with ethical approval from the Fourth Hospital of Hebei Medical University (no. 2024KS055) and patient consent. These organoids were subsequently cultured using the Esophagus Cancer Organoid Kit (BioGenous, K2177-ES) and Matrigel (BD Bioscience). The ESCC organoids were distributed into a 96-well plate, with each well containing 3,000 cells and 7 μl of Matrigel after resuspension in a gel matrix. After a period of 3 d, the organoids were exposed to varying concentrations of CMSP. Bright-field images were captured for each well 48 h after treatment. The surface area of the ESCC organoids (μm^2^) was measured using ImageJ software. Subsequently, the viability of the surviving organoids was assessed using the Cell Counting Kit-8 (CCK-8) assay (Solarbio, CA1210-1000), which is a sensitive assay based on mitochondrial activity. Inhibition scores were calculated by comparison with vehicle-treated wells.

### GFP-LC3B or mRFP-GFP-LC3B assay

For the investigation of autophagy, cells were transfected with GFP-LC3B or mRFP-GFP-LC3B plasmids (2 μg) for 24 h and then treated with various chemicals for 12 h. After fixing with 4% paraformaldehyde in phosphate-buffered saline (PBS), confocal microscopy (Nikon, Japan) was employed to capture images. Autophagy regulators were identified by exposing cells to different substances for 12 h and counting the average GFP-LC3B puncta per cell. Autophagic flux was assessed by counting GFP^+^ mRFP^+^ (yellow) and GFP^−^ mRFP^+^ (red) LC3B puncta using confocal microscopy, with at least 5 representative fields counted per experiment.

### TEM assay

KYSE-30 and KYSE-150 cell lines underwent the specific treatment and were then fixed in 4% glutaraldehyde at 4 °C overnight. Following dehydration, ultrathin sections were embedded and stained with uranyl acetate and lead citrate. Imaging was conducted using a transmission electron microscope (HITACHI, Japan).

### Quantitative proteome analysis

For proteome analysis, KYSE-30 cells were treated with DMSO or CMSP for 48 h, after which cell samples were collected for analysis via HPLC-MS/MS (Thermo Fisher, USA). The quantitative proteomic analysis involved 4 sequential steps: protein extraction, sample preparation using filter-aided sample preparation (FASP), peptide fractionation, and MS analysis. Proteome Discoverer 2.2 processed the MS/MS raw data using a reverse database search to ensure a 1% false discovery rate for peptide and protein. Proteins exhibiting a fold change greater than 2, with the *P* value less than 0.05, were identified as differentially expressed.

### ECAR and OCR

The ECAR and OCR of KYSE-30 and KYSE-150 cells were quantified utilizing the Seahorse XF extracellular flux analyzer (Agilent, USA). The cells underwent treatment with specified concentrations of CMSP for a duration of 36 h. Following this, the cells were seeded at 1 × 10^4^ per well in 96-well Seahorse plates and allowed to adhere overnight in growth medium. Subsequently, the adherent cells were washed and fresh assay medium was added before analysis. The cartridge was prepared to dispense glucose, oligomycin, and 2-deoxy-D-glucose (2-DG) for ECAR measurement, and oligomycin, carbonyl cyanide 4-(trifluoromethoxy) phenylhydrazone (FCCP), antimycin A, and rotenone for OCR measurement. The ECAR and OCR values were recorded and analyzed using the Seahorse XF96 software.

### Cellular target identification of CMSP

KYSE-30 cell lysates were incubated with CMSP or biotin-CMSP overnight at 4 °C and then treated with *Streptavidin* beads for 6 h. After 6 washes with a buffer containing 1% NP40 and 200 mM NaCl, CMSP-binding proteins were isolated using sodium dodecyl sulfate–polyacrylamide gel electrophoresis (SDS-PAGE) and identified by silver staining assay. Protein bands with significant changes from biotin-CMSP treatment were excised, digested with trypsin, and analyzed by LC-MS/MS (Thermo Scientific, USA).

### Molecular docking and molecular dynamics analysis

The LDHA structure [Protein Data Bank (PDB) ID: 4ZVV] was obtained from the Research Collaboratory for Structural Bioinformatics Protein Data Bank (RCSB PDB). Autodock and PyMOL software were used for molecular docking and visualization of CMSP–LDHA interactions.

### Cellular thermal shift assay

The verification of the cellular target of CMSP was conducted utilizing CETSA, as outlined in prior studies [64]. Briefly, cells were exposed to CMSP or left untreated for a duration of 4 h, subsequently subjected to heating at various temperatures ranging from 46 to 62 °C for 3 min, and then cooled on ice. The soluble lysates were centrifuged, and the supernatants were analyzed by Western blotting.

### Biolayer interferometry assay

The binding affinity of CMSP to LDHA was assessed using a BLI assay on the Octet RED96 system (ForteBio, CA, USA). His-LDHA was immobilized on Ni-NTA biosensor tips (ForteBio, CA, USA) following pre-equilibration with a kinetic buffer consisting of PBS, 0.05% bovine serum albumin (BSA), and 0.01% Tween 20. His-LDHA was loaded onto equilibrated Ni-NTA biosensors at 100 μg/ml. The assays were executed in accordance with standard protocols using 96-well black plates, with a total volume of 200 μl per well, maintained at a temperature of 30 °C. Data analysis was performed using the Octet data analysis software, which incorporated a double reference subtraction protocol to account for nonspecific binding, background signals, and signal drifts resulting from biosensor variability. The equilibrium dissociation constant (*K*_d_) was calculated as the ratio of the off-rate constant (*K*_off_) to the on-rate constant (*K*_on_).

### His-tag pull-down assay

Following the manufacturer’s guidelines, purified His-tagged LDHA protein was incubated with Dynabeads His-tag isolation magnetic beads (10103D, Invitrogen) in a binding buffer at 4 °C for 4 h. After removing unbound proteins, the protein-coupled beads were incubated with purified flag-FGFR1, with or without CMSP, for an additional 4 h in a pull-down buffer at 4 °C. The beads underwent 5 washes using the binding buffer. Ultimately, the beads were boiled in dithiothreitol (DTT) and analyzed through Western blot.

### Membrane protein extraction

KYSE-30 cells were cultured in the presence of either DMSO or 40 μg/ml CMSP for 48 h, followed by membrane protein isolation using the Mem-PER Plus Kit (89842, Invitrogen), according to the manufacturer’s instruction. The resulting cytosolic and plasma membrane fractions were subjected to Western blotting, employing GAPDH and sodium potassium adenosine triphosphatase (ATPase) as loading controls for the cytoplasmic and plasma membrane fractions, respectively.

### Metabolomics profiling

KYSE-30 cells were cultured in 10-cm dishes and exposed to DMSO or 40 μg/ml CMSP for 36 h. After treatment, cells were washed thrice with cold PBS, collected, and flash-frozen in liquid nitrogen for 15 min to prepare for subsequent LC-MS/MS analysis (Wuhan Metware Biotechnology Co. Ltd.).

### Mouse xenograft models

Mice were kept in pathogen-free conditions, with experiments approved by the Fourth Hospital of Hebei Medical University’s Bioethics Committee (IACUC-4th Hos Hebmu-2022001). To establish the cell-derived xenograft mouse model, BALB/c nude mice (HFK Bioscience Co. Ltd., Beijing, China) were subcutaneously injected with 1 × 10^6^ KYSE-30 cells. The mice were then randomly assigned to 1 of 4 groups: (a) Control, (b) 10 mg/kg CMSP, (c) 30 mg/kg CMSP, (and d) 30 mg/kg CMSP + 2 mg/kg rapamycin. Once tumor volumes reached approximately 50 mm^3^, CMSP or vehicle (5% DMSO in PBS) was given by intraperitoneal administration every 3 d. Tumor volume was calculated as 0.5 × length × width^2^. At the termination, the mice were euthanized and the tumors were collected. The blood and tumors from the mice within this study were harvested and used for in vitro assays.

### Statistical analysis

SPSS software and GraphPad Prism were used for statistical analysis. The data are presented as mean ± SD from at least 3 independent experiments. Statistical significance was calculated by 2-tailed Student’s *t* test, chi-square test, or one-way analysis of variance (ANOVA), with significance levels denoted as *P < 0.05 and **P < 0.01.

The additional methods and materials are provided in the Supplementary Materials.

## Data Availability

The proteomics data from MS have been submitted to the ProteomeXchange Consortium through the iProX partner repository with the dataset identifier PXD060760.
