# Supplementary material for: A Novel Autophagy Inhibitor p-Hydroxylcinnamaldehyde Suppresses Esophageal Squamous Cell Carcinoma by Targeting LDHA Phosphorylation-Mediated Metabolic Reprogramming
Source: Research (Wash D C). 2026 Jan 12;9:1070. doi: 10.34133/research.1070 (PMC12794202; doi:10.34133/research.1070)
Supplement: Supplementary 1 — Materials and Methods Figs. S1 to S6 Tables S1 to S3 [file research.1070.f1.zip › Supplementary information.docx]

**Supplementary Information**


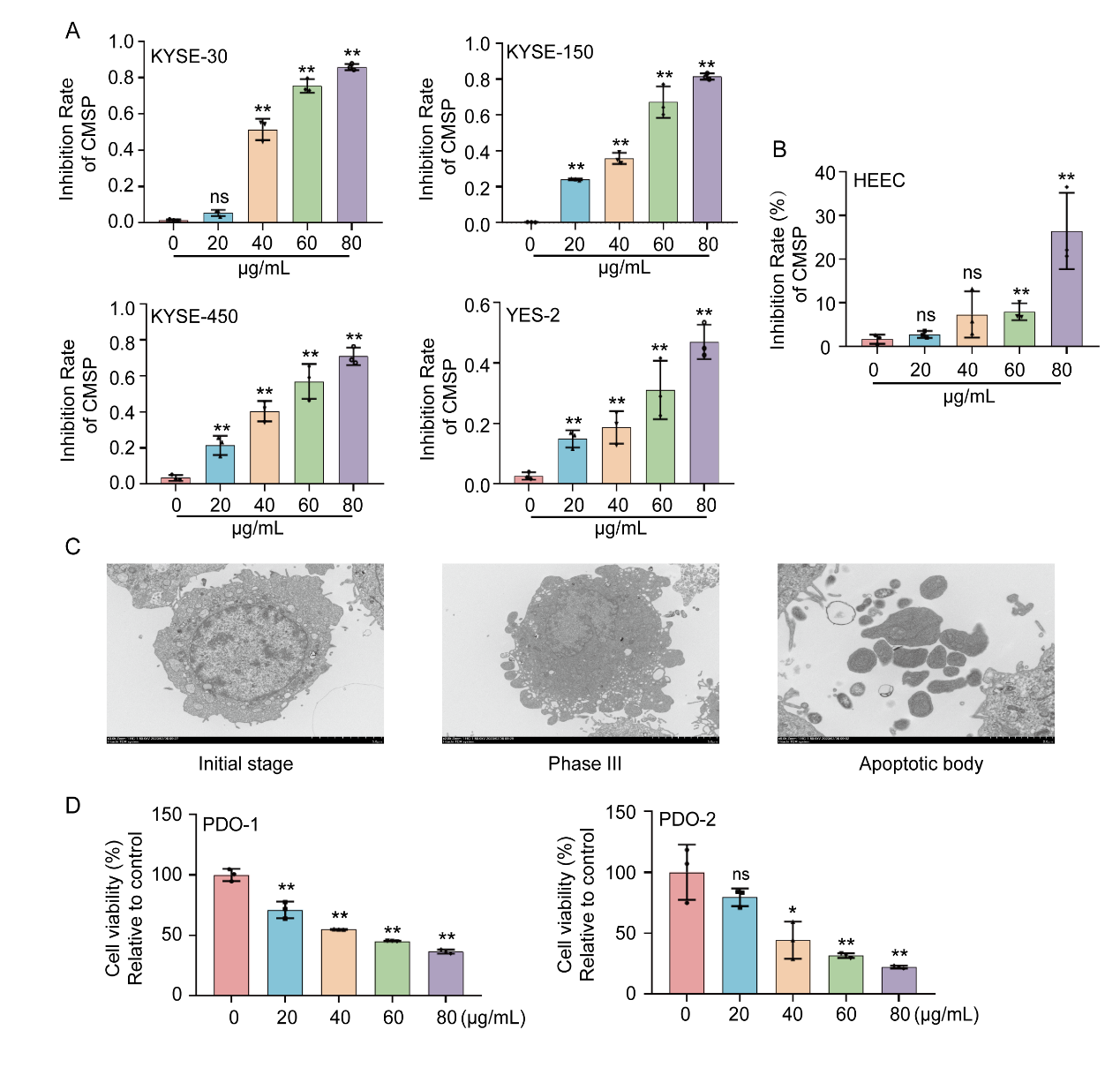


**Figure S1 CMSP induces apoptosis of ESCC cells.** A, B) ESCC cell lines (A) and HEEC (B) were treated with various concentrations of CMSP and DMSO (< 0.01%) for 36 h. Cell viability was determined by MTS assay. Data are shown as the mean ± SD. C) Representative electron micrographs of KYSE-30 treated with CMSP. D) Cell viability data were analyzed and plotted relative to untreated organoids. Data are shown as the mean ± SD (n = 3). **p* < 0.05 and ***p* < 0.01 *versus* the control group.


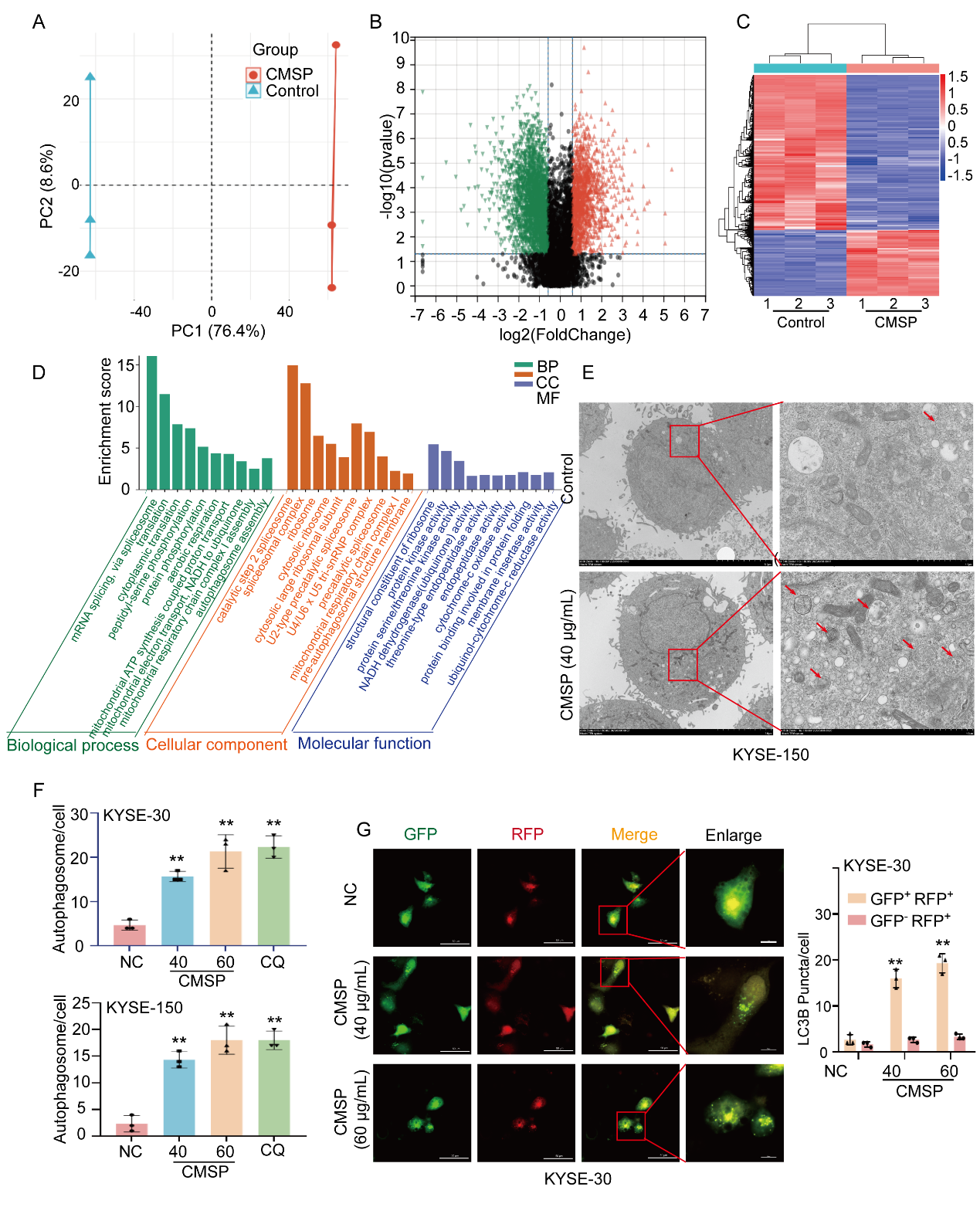


**Figure S2 Quantification of Proteomic profiles in KYSE-30 cells treated with CMSP.** A) PCA of proteomics with or without CMSP treatment represented in a two-dimensional space. B) Volcano plot showed the differentially expressed proteins between CMSP-treated and untreated KYSE-30 cells. C) The hierarchical clustering analysis and heatmap of the correlation coefficients between protein expression profiles. D) GO analysis for the significantly differentially expressed proteins CMSP compared to control in KYSE-30 cells. E) KYSE-150 cells were treated with CMSP (40 μg/mL) for 36 h, fixed and examined using transmission electron microscopy. Higher power magnification of the image of CMSP-treated cells revealed autophagosomes (arrows).

F) The statistical data of CMSP on GFP-LC3B punctation. G) Confocal microscopy showing the yellow LC3B (autophagosome) and red LC3B (autolysosome) dots per cell in each condition of KYSE-30. Data are shown as the mean ± SD (n = 3). ***p* < 0.01 *versus* the control group.


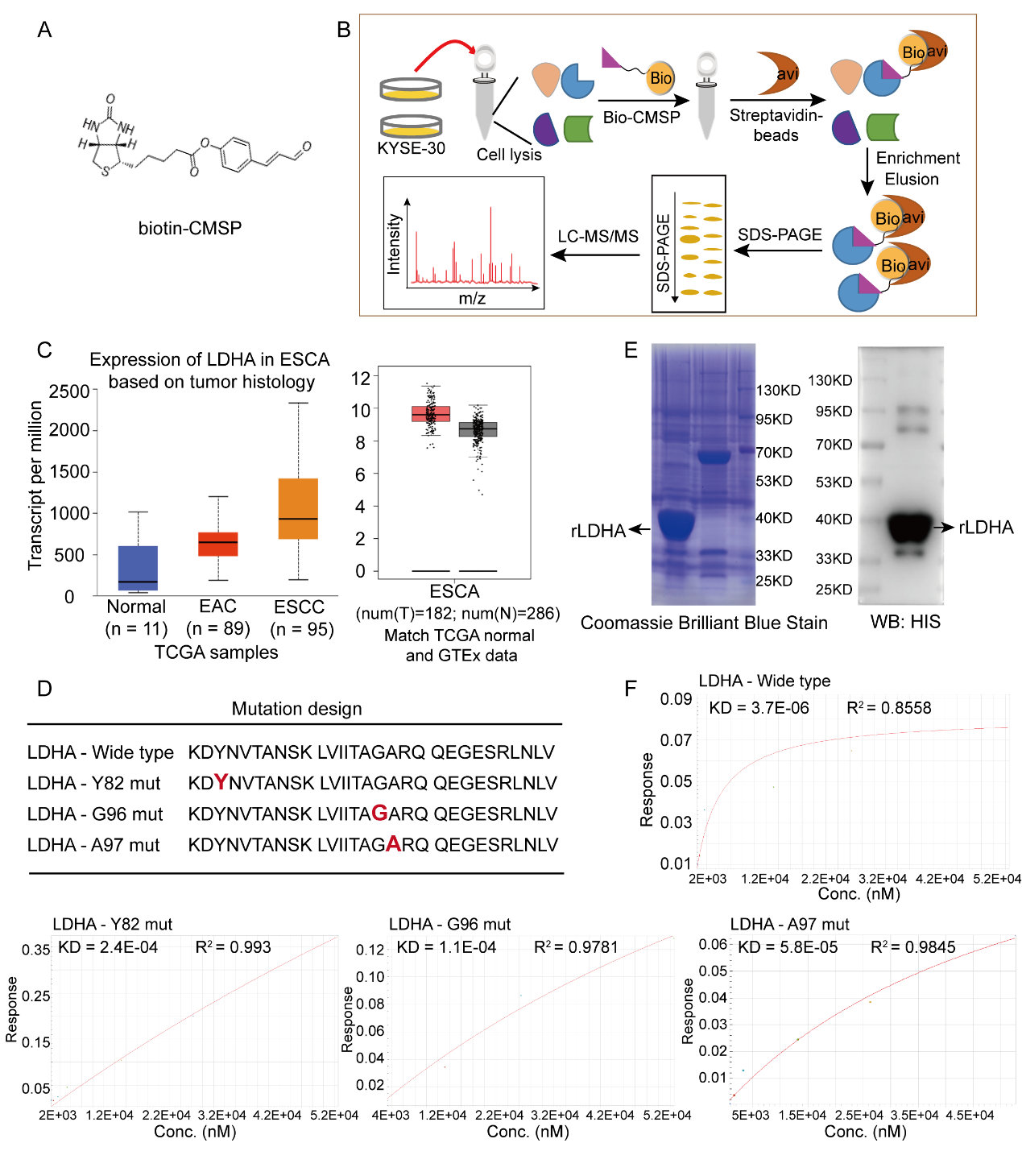


**Figure S3 CMSP directly targets LDHA.** A) Chemical structure of biotin-CMSP. B) Schematic process for the identification of CMSP specific binding targets. C) The expression of LDHA in ESCA base on tumor histology in TCGA. D) Diagram showing the mutation design. E) Validation of purified recombinational LDHA protein by Coomassie brilliant blue stain and western blot. F) The steady state of CMSP to recombinant LDHA and mutation protein.


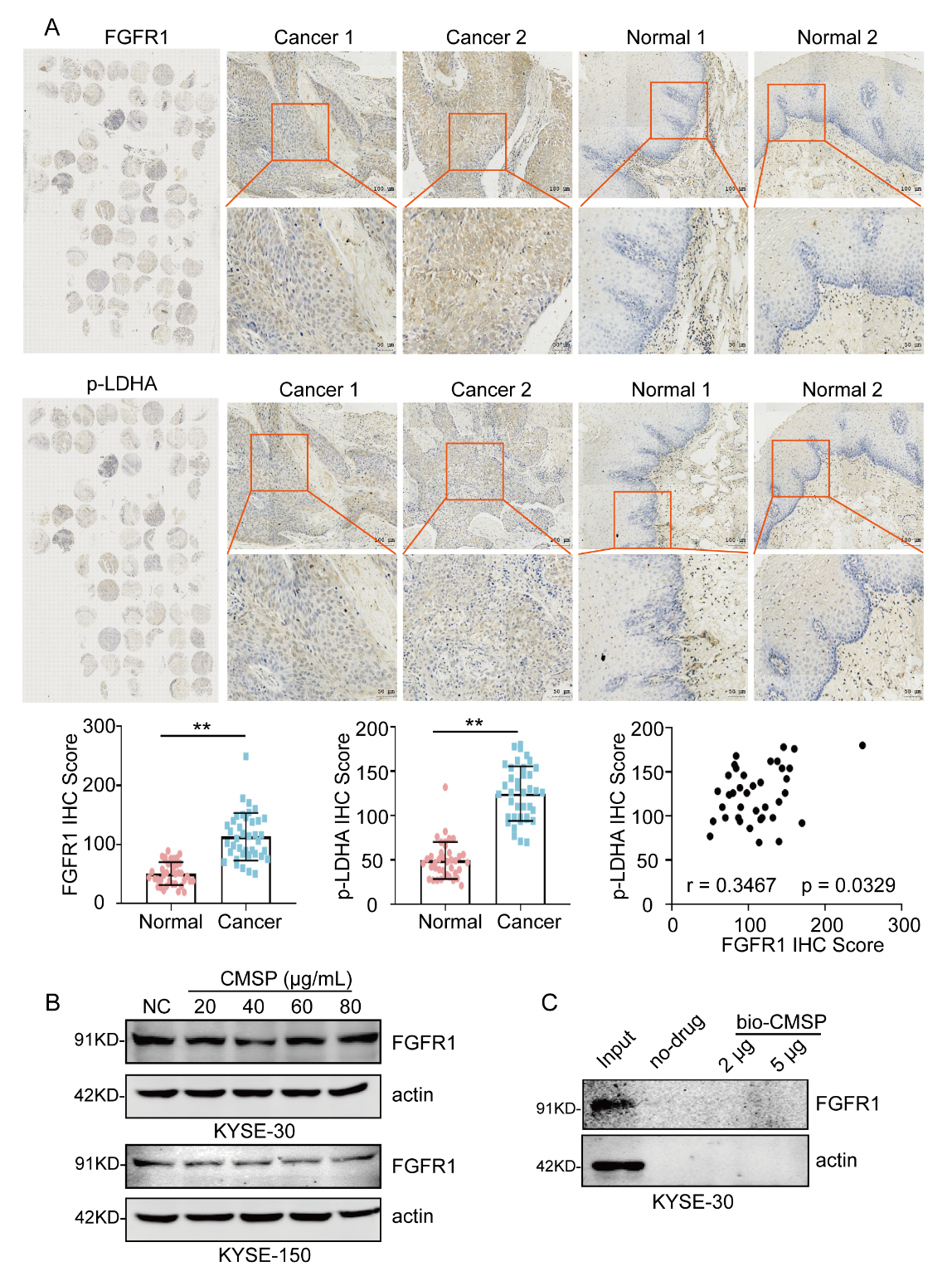


**Figure S4 CMSP blocks LDHA phosphorylation through disconnection with FGFR1.** A) The expression and correlation of FGFR1 and p-LDHA in ESCC microarrays. B) The effect of CMSP on expression of FGFR1 by western blot. C) Validation of the interaction between FGFR1 and CMSP. Data are shown as the mean ± SD (n = 3). ***p* < 0.01 *versus* the control group.


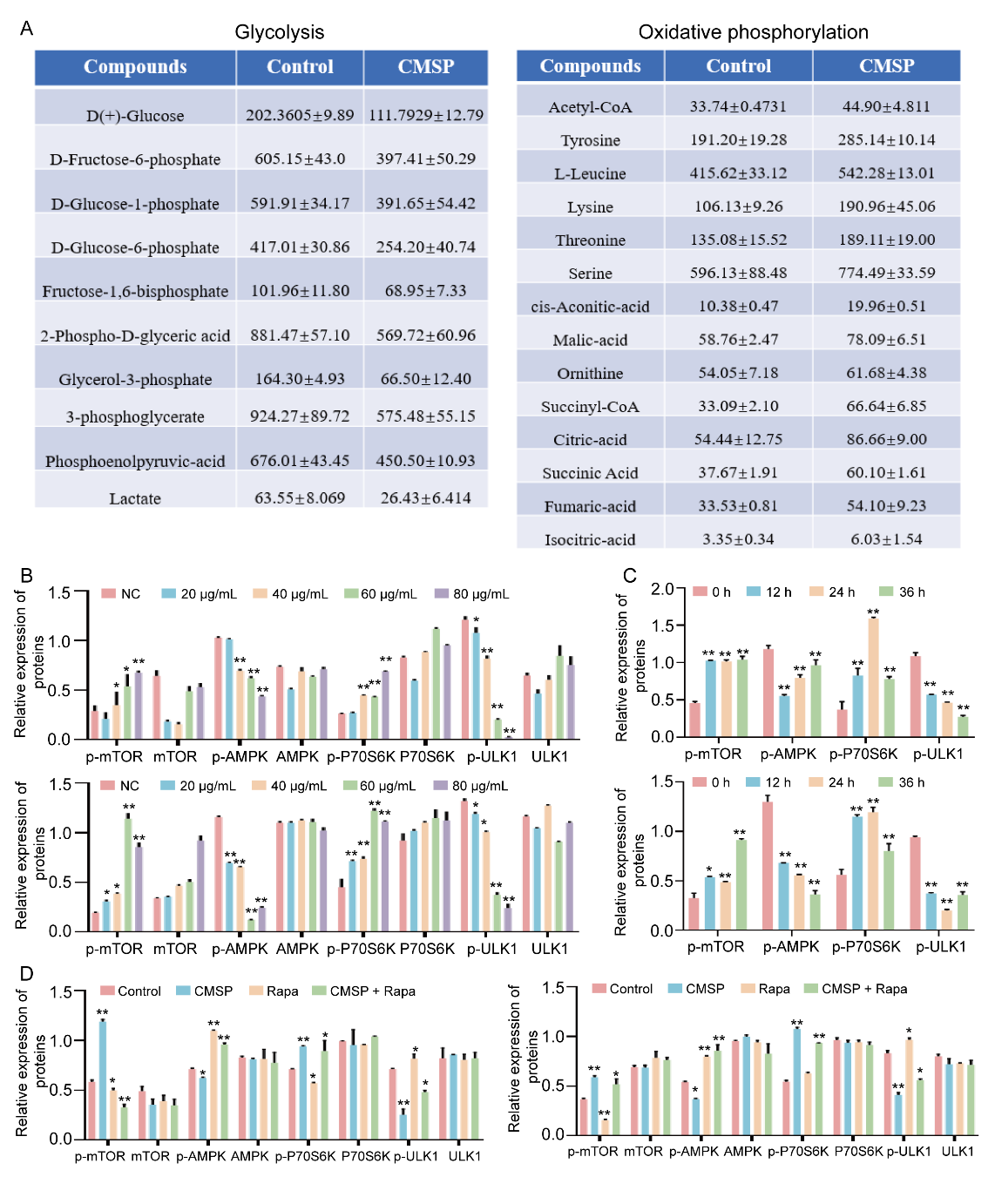


**Figure S5 Metabonomic analysis of CMSP on ESCC cells.** A) Metabonomic analysis showing the critical metabolites of glycolysis and oxidative phosphorylation following CMSP treatment. B-D) Statistical analysis on the relative expression of proteins. Data are shown as the mean ± SD (n = 3). **p* < 0.05, ***p* < 0.01 *versus* the control group.


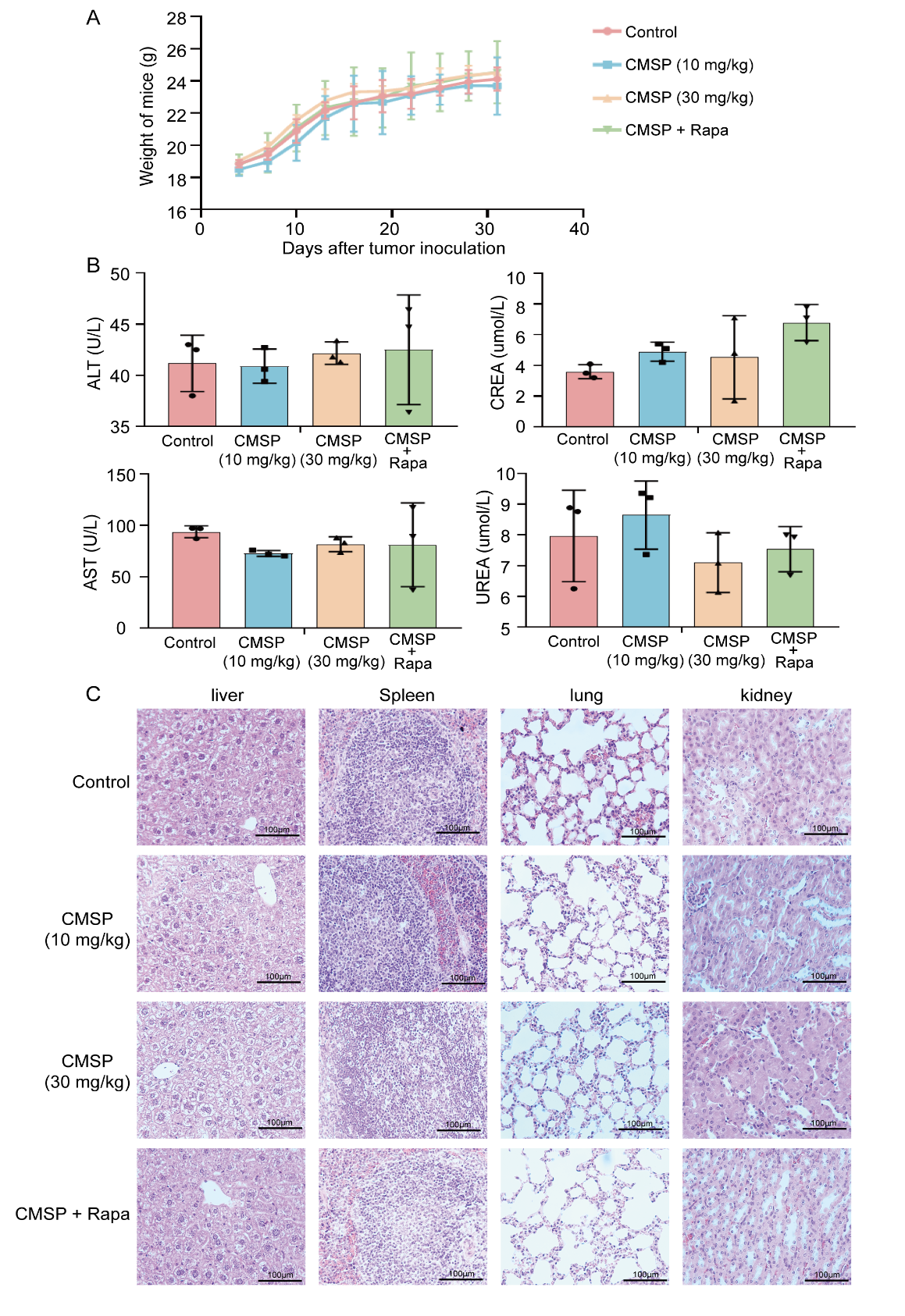


**Figure S6 Evaluation of *in vivo* side effect of CMSP.** A) Body weight curves of mice in different groups during the experimental period. B) Hepatic (ALT and AST) and renal (UREA and CREA) function of nude mice after the last drug administration. All data were from three repeats. C) Histological analysis of major organs (liver, spleen, lung, and kidney) from nude mice.
