# Supplementary material for: A Novel Autophagy Inhibitor p-Hydroxylcinnamaldehyde Suppresses Esophageal Squamous Cell Carcinoma by Targeting LDHA Phosphorylation-Mediated Metabolic Reprogramming
Source: Research (Wash D C). 2026 Jan 12;9:1070. doi: 10.34133/research.1070 (PMC12794202; doi:10.34133/research.1070)
Supplement: Supplementary 1 — Materials and Methods Figs. S1 to S6 Tables S1 to S3 [file research.1070.f1.zip › Supplementary materials.docx]

**Additional Material and methods**

**2.18 Annexin Ⅴ apoptosis assay**

KYSE-30 or KYSE-150 cells were seeded onto 6 cm culture dishes, achieving a cell confluence of 70%. After a 16-hour incubation period, varying concentrations of CMSP, dissolved in RPMI-1640 medium, were administered to each dish for a duration of 48 h. Subsequently, the cells were harvested, washed three times with ice-cold PBS, and stained with annexin Ⅴ and 7-AAD (BD Pharmingen). Fluorescence were quantified using a flow cytometer (BD, San Diego, CA, USA).

**2.19 Organoid immunofluorescence assay**

Cultured ESCC organoids with or without CMSP (60 μg/mL) treatment were collected and fixed in 4% paraformaldehyde for 30min at 4 °C, washed with PBS, and permeabilized with 0.25% Triton X-100 in PBS for 20 min. The organoids were then washed with PBST (PBS containing 0.1% Tween 20) and blocked by 5% BSA in PBST for 1 h at room temperature. Organoids were incubated with the primary antibodies at 4 °C overnight, washed with PBST for three times, and then incubated with the secondary antibodies for 1 h at room temperature in the dark, finally, stained with DAPI for 15 min in the dark. Organoid imaging was performed on confocal microscope.

**2.20 Western blot**

Proteins were extracted from the cells using RIPA buffer (Solarbio, China) supplemented with protease and phosphatase inhibitors (Roche, Switzerland). The resulting mixture was centrifuged at 12000 rpm for 10 min at 4 ℃. The total protein concentration was quantified using the BCA assay (Thermo Scientific, USA). Subsequently, the protein samples underwent SDS-PAGE and were transferred onto a PVDF membrane (Millipore, USA). The membranes were blocked with 5% skim milk at room temperature for 1 h, followed by an overnight incubation with primary antibodies at 4 ℃. Afterwards, the membranes were incubated with a secondary antibody, either fluorescence-conjugated anti-rabbit or anti-mouse IgG, at room temperature for 1 h. Visualization of the membranes was performed using an Odyssey assay (Millipore, USA).

**2.21 Quantitative proteome analysis**

For proteome analysis, KYSE-30 cells were treated with DMSO or CMSP for 48 h, and cell samples were collected for analysis *via* HPLC-MS/MS (Thermo Fisher, USA). The quantitative proteome analysis involved four sequential steps: protein extraction, sample preparation using filter-aided sample preparation (FASP), peptide fractionation, and mass spectrometry analysis. MS/MS raw data were processed using Proteome Discoverer 2.2, with a peptide and protein false discovery rate set at 1% through a reverse database search strategy. Proteins exhibiting a fold change greater than 2 and *p* value less than 0.05 were classified as differentially expressed.

**2.22 Detection for lactate**

The content of lactate was detected by commercially available kits (Solarbio, China) according to the instructions.

**2.23 Immunohistochemistry**

The tumor tissues from the *in vivo* experiment were fixed with 4% formaldehyde for at least 48 h and then enclosed within paraffin blocks. The paraffin sections were deparaffinized at 60 ℃ for 2 h and then rehydration using alcohol and PBS. Next, the slides were boiled in sodium citrate buffer solution for 3 min. Afterwards, the tissue sections were treated with 3% H_2_O_2_ for 20 min and then blocked in 10% goat serum for 1 h at room temperature. The slides were subsequently incubated with the following antibodies (Ki67, 1:2000; LC-3B, 1:200; cleaved caspase 3, 1:500) at 4 ℃ overnight. The slides were then washed with PBS and stained with the appropriate secondary antibody. Finally, the slides were stained with diaminobenzidine and then counterstained with haematoxylin. Representative fields of each slide were photographed using an inverted microscope.

**2.24 LysoTracker Red staining**

Tumor cells were exposed to specific chemical treatments for 48 h at 37 ℃, followed by an additional 1 h incubation with 50 nM LysoTracker Red (Beyotime, China). Then, the cells were fixed with 4% paraformaldehyde and subjected to DAPI staining for 10 min. The samples were then analyzed using a confocal microscope (Nikon, Japan).

**2.25 Acridine orange (AO) staining**

AO is a fluorescent dye known for its ability to bind to nucleic acids and proteins in pH-dependent. In acidic environments, such as lysosomal compartments, AO exhibits red fluorescence with an emission peak around 650 nm. Conversely, in neutral or alkaline environments, such as cytosolic and nuclear compartments, AO displays green fluorescence with an emission range between 530 and 550 nm. Therefore, AO fluorescence will be as an indicator for detecting changes in lysosomal acidity and integrity. Tumor cells were incubated with specified compounds for 48 h or subjected to starvation using EBSS. AO (ACROS, Belgium) staining was conducted at concentration of 5 μg/mL for 30 min under condition of 37 ℃ and 5% CO_2_. Following three washes with PBS, images were acquired using confocal microscope (Nikon, Japan).
